# Using quantitative PCR with retrotransposon-based insertion polymorphisms as markers in sugarcane

**DOI:** 10.1093/jxb/erv283

**Published:** 2015-06-19

**Authors:** Cushla J. Metcalfe, Sarah G. Oliveira, Jonas W. Gaiarsa, Karen S. Aitken, Monalisa S. Carneiro, Fernanda Zatti, Marie-Anne Van Sluys

**Affiliations:** ^1^GaTE-Lab, Departamento de Botânica, IBUSP, Universidade de São Paulo, rua do Matao 277, 05508-090, SP, Brazil; ^2^CSIRO Agriculture Flagship, Queensland Bioscience Precinct, 306 Carmody Road, St Lucia, QLD 4072, Australia; ^3^Centro de Ciências Agrárias, Universidade Federal de São Carlos, Araras, 13600-970, SP, Brazil

**Keywords:** Marker, polyploid, real-time PCR, retrotransposon-based insertion polymorphism, sugarcane, transposable element.

## Abstract

Sugarcane is the main source of the world’s sugar and is becoming increasingly important as a source of biofuel. The highly polyploid and heterozygous nature of the sugarcane genome has meant that characterization of the genome has lagged behind that of other important crops. Here we developed a method using a combination of quantitative PCR with a transposable marker system to score the relative number of alleles with a transposable element (TE) present at a particular locus. We screened two genera closely related to *Saccharum* (*Miscanthus* and *Erianthus*), wild *Saccharum*, traditional cultivars, and 127 modern cultivars from Brazilian and Australian breeding programmes. We showed how this method could be used in various ways. First, we showed that the method could be extended to be used as part of a genotyping system. Secondly, the history of insertion and timing of the three TEs examined supports our current understanding of the evolution of the *Saccharum* complex. Thirdly, all three TEs were found in only one of the two main lineages leading to the modern sugarcane cultivars and are therefore the first TEs identified that could potentially be used as markers for *Saccharum spontaneum.*

## Highlight

qPCR-RBIP was used to examine the dosage of particular markers and evolutionary history in *Saccharum* and the related genera, *Erianthus* and *Miscanthus*. It also differentiated between *S. spontaneum* and *S. officinarum*.

## Introduction

The search for biofuels, i.e. fuels produced from renewable biofeedstocks or biomass, has been driven by concerns about rising prices, limited supplies of petroleum, and the effect of greenhouse gases (Hill *et al.*, 2006; Zinoviev *et al.*, 2010). The world’s principal biofuel is bioethanol fermented from sugars (sucrose and starch). The USA and Brazil together account for more than 87% of the world’s bioethanol production; in the USA, bioethanol is derived from fermentation of corn grain starch, while in Brazil it is derived from sugarcane juice and molasses (Botha and Moore, 2014). Bioethanol is a first-generation biofuel, i.e. a fuel produced from plant energy storage molecules such as sugars, starch, and lipids (Chong and O’Shea, 2012). Concerns that using food crops as biofuels could drive up food costs, as well as high production and processing costs, have led to interest in second-generation biofuels (Sims *et al.*, 2010). These are derived from lignocellulose or fibre, i.e. waste material from food production, agricultural residues, or dedicated cellulosic crops (Chong and O’Shea, 2012). It has been suggested that, in order to meet demand, second-generation biofuels cannot rely solely on production from waste, and that it is necessary to develop dedicated cellulosic crop, which can be grown on substandard soil (Chong and O’Shea, 2012). Sugarcane as a dedicated cellulosic crop was first described by Alexander (1985) and was termed ‘energy cane’. Energy cane can be defined as a sugarcane variety bred for higher fibre levels than traditional sugarcane varieties, which have been bred for high sucrose content and some fibre (Matsuoka *et al.*, 2014).

**Fig. 1.**
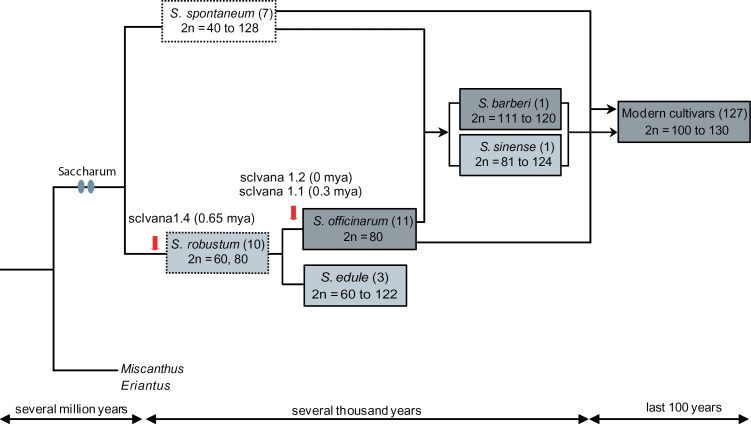
Evolutionary history of *Saccharum* species modified from Grivet *et al.* (2006). Solid and dashed lines with arrows indicate hybridization events and minor contributions to modern sugarcane cultivars, respectively. Grey ovals show whole genome duplication events. 2*n* refers to chromosome numbers from Grivet *et al.* (2006). Red arrows show the timing of insertion of the scIvana element. The numbers in brackets after the scIvana element indicates the estimated age of the insertion of the element (mya, million years ago). Outline of the box and box colour indicate type of sugarcane and number of scIvana elements identified, respectively; dashed outline, wild species; solid outline, domesticated species; white, no scIvana element found; pale grey scIvana 1.4 only found; grey, scIvana 1.4 and 1.2 found; dark grey all 3 scIvana elements found.The number in brackets after the species name indicates the number of samples examined. (This figure is available in colour at *JXB* online.)

Modern sugarcane cultivars are highly polyploid or aneuploid hybrids derived from interspecific hybridization between *Saccharum officinarum* and *Saccharum spontaneum*, a wild sugarcane (Moore *et al.*, 2014) (Fig. 1). *S. officinarium* and *S. spontaneum* have contrasting attributes in terms of sucrose and fibre content: *S. officinarium* has high sucrose and low fibre content, while *S. spontaneum* has low sucrose and high fibre content (Moore *et al.*, 2014). Hybrids between *S. officinarum* and *S. spontaneum* show 2*n*+*n* transmission, where 2*n* is the entire genome of *S. officinarum*. This phenomena remains true in the first backcross between the 2*n*+*n* F1 and the female *S. officinarum*, but generally breaks down in subsequent backcrosses (Bremer, 1961; Piperidis *et al.*, 2010). Early breeders used this phenomenon to introduce vigour and resistance genes from *S. spontaneum*, while quickly recovering the high sugar content of *S. officinarum* (Roach, 1972). The modern sugarcane cultivar has chromosome numbers ranging from 100 to 120, 70–80% of which are from *S. officinarum*, 10–23% from *S. spontaneum*, and a small portion being recombinants (D’Hont, 2005; Piperidis *et al.*, 2010). The number of alleles most likely varies from 8 to 14 (Aitken *et al.*, 2014b). For almost all cultivars the genome size is unknown; the modern R570 cultivar has a genome size of ~10 Gb (D’Hont and Glaszmann, 2001). Current molecular evidence suggests that *S. officinarum* itself is derived from the wild sugarcane, *Saccharum robustum* (Lu *et al.*, 1994; D’Hont *et al.*, 1998). The older traditional cultivars *Saccharum barberi* (North India) and *Saccharum sinense* (China) are thought to be natural hybrids (D’Hont *et al.*, 2002).

Today, there are a number of breeding programmes attempting to breed energy cane (Chong and O’Shea 2012). Traditional breeding programmes, i.e. programmes breeding for high-sucrose cultivars, use crosses between commercial or near-commercial cultivars (Matsuoka *et al.*, 2014), which have been shown to be genetically very similar (Dal-Bianco *et al.*, 2012). The development of a new cultivar is also time and resource intensive: it takes at least 250 000 seedlings and 12–15 years to create a commercially viable cultivar in traditional breeding programmes (Hotta *et al.*, 2010; Zhang *et al.*, 2014). To broaden the genetic basis of sugarcane and develop an energy cane with higher biomass, several breeding programmes are including ancestral genotypes, i.e. *S. spontaneum* and *S. robustum*, in crosses (Wang *et al.*, 2008; van der Weijde *et al.*, 2013; Santchurn *et al.*, 2014). In order to speed up selection of yield and desirable traits, research must continue to decipher the complex sugarcane and develop makers for marker-assisted selection (Waclawovsky *et al.*, 2010).

The use of new technologies for genome-assisted selection and genetic improvement has lagged behind in sugarcane due to the polyploid and heterozygous nature of its genome. Several initiatives have recently been published, which took advantage of the high throughput and reduced cost of new sequencing strategies to improve our knowledge of sugarcane genomics (Garcia *et al.*, 2013; Berkman *et al.*, 2014; Cardoso-Silva *et al.*, 2014; de Setta *et al.*, 2014; Grativol *et al.*, 2014; Nishiyama *et al.*, 2014). Next-generation sequencing of sugarcane cultivars and wild *Saccharum* species has provided information regarding repetitive content and allelic variation (Berkman *et al.*, 2014), a bacterial artificial chromosome (BAC)-based sequencing of 3.7% of the monoploid genome established the framework for genomic annotation and for evolutionary studies with closely related Poaceae species (de Setta *et al.*, 2014). Grativol *et al.* (2014) revealed the methylation landscape of the sugarcane genome using a methylfiltration sequencing approach. Transcript assemblies from contrasting sugarcane varieties and full-length cDNA sequences have provided complimentary information on gene expression profiles, and have resulted in an improved understanding of gene structure and the regulatory environment (Cardoso-Silva *et al.*, 2014; Nishiyama *et al.*, 2014).

The complexity of the sugarcane genome makes the use of molecular markers very difficult. The use of simplex makers (markers that segregate 1:1 in progeny from a biparental cross or 3:1 in selfed progeny; D’Hont *et al.*, 2010) was first described by Ritter *et al.* (1990) and refined by Wu *et al.* (1992) for polyploids. In the last few decades, there has been a great deal of progress in using simplex markers for diversity analysis, analysis of genetic relationships, genetic linkage mapping, mapping of simply inherited traits and complex traits, and association mapping (reviewed by Henry and Kole, 2010). Markers used include restriction fragment length polymorphisms (Ming *et al.*, 2001, 2002), randomly amplified polymorphic DNA (Nair *et al.*, 1999), microstatellites, and simple sequence repeats (Cordeiro *et al.*, 2003; Pinto *et al.*, 2004), amplified fragment length polymorphisms (Aitken *et al.*, 2006), and target region amplification polymorphisms (Devarumath *et al.*, 2013). These marker systems have been used to create genetic linkage maps (Aitken *et al.*, 2005, 2014b), identify quantitative trait loci associated with disease resistance, sugar content, and stalk attributes (reviewed by Pastina *et al.*, 2010), identify relationships within *Saccharum* species (Nair *et al.*, 1999), assess genetic diversity within *Saccharum* species (Aitken *et al.*, 2006; Alwala *et al.*, 2006; Arro *et al.*, 2006), and carry out molecular genotyping of cultivars (Pan *et al.*, 2007; Parida *et al.*, 2009).

Here, we describe the development of a marker system that can estimate the dosage of a particular marker. This system is based on TaqMan quantitative PCR (qPCR) combined with retrotransposon-based insertion polymorphism (RBIP). RBIP is a PCR marker system that identifies the insertion of a type of transposable element (TE), long terminal repeat retrotransposons (LTR-RTs), which, as their name suggests, have flanking terminal repeats (Wicker *et al.*, 2007). We chose the RBIP system because LTR-RTs are ubiquitous and usually present in high copy numbers; they are widely dispersed throughout the genome and show insertional polymorphism within and between plant species (Kumar and Hirochika, 2001). LTR-RTs create a target site duplication (TSD), a short direct repeat that is generated on both flanks of a TE upon insertion. TSD length, but not sequence, is characteristic of a particular TE superfamily. The TSD plus flanking sequence can therefore be used to identify individual TE insertions. Because of their mode of replication, most insertions are irreversible, so they can be used to determine parental lineage or introgression. Finally, it is possible to date the time of insertion of a single LTR-RT element by the nucleotide divergence of its LTRs (Ma *et al.*, 2004). This allows us to examine the evolutionary history of individual LTR-RTs or the timing of divergence of taxa, based on the timing of the appearance of elements in a taxon.

Seven LTR-RT families have been described previously in sugarcane (Domingues *et al.*, 2012). We chose to examine three scIvana elements because unpublished results using the PCR-based RBIP strategy suggested that scIvana insertions are highly polymorphic among sugarcane cultivars/species and that the number of alleles at a single locus with the scIvana present is highly variable (Domingues, 2009). ScIvana elements are 5–5.9kb in total length, with LTRs of about 240–450bp, and are present in low copy number (Domingues *et al.*, 2012). Here, we combined qPCR with RBIP to estimate the ratio of alleles with the scIvana present at three loci (three different insertions of a scIvana element). Because of the complex polyploid nature of the sugarcane genome, the number of alleles and genome size for most clones and species is unknown. We therefore used a relative quantitative method using the relative cycle threshold (*C*_t_) values of the two reactions, one designed to detect the presence of the element and the other designed to detect the absence of the element, at a particular locus, to estimate the ratio of the number of alleles with the scIvana present.

A dosage marker system could be used like any other maker, i.e. as a molecular genotyping system, to estimate genetic diversity or as a marker associated with a trait. Features of LTR-RTs also allow us to trace the appearance of elements and the evolutionary history of taxa, as described above. The use of qPCR rather than PCR enabled us to estimate the relative dosage of alleles with the element present. Using qPCR to estimate relative allele dosage could be extended to other genomic elements such as genes of interest. We first showed that the qPCR-RBIP system was replicable. We were unable to link the ratio of the number of alleles with the scIvana present to any particular trait but did demonstrate that the system could be used as part of a cultivar genotyping system. We then used the qPCR-RBIP strategy to examine the evolutionary history of three scIvana elements in *Saccharum* and two closely related genera, *Erianthus* and *Miscanthus*. We also showed that the method can be used as a potential marker to differentiate *S. spontaneum* and *S. officinarum*, the ancestral genotypes that are currently in use in several sugarcane breeding programmes for the selection of energy canes (Wang *et al.*, 2008; van der Weijde *et al.*, 2013; Santchurn *et al.*, 2014).

## Materials and methods

### Samples and genomic DNA extraction

A list of a cultivars and species used and their parents, provenance, and traits, where known, is given in Supplementary Table S1 (available at *JXB* online).

Nine species from the two genera closely related to *Saccharum* were examined, five *Miscanthus* and four *Erianthus* species. Of the wild *Saccharum* and traditional cultivars, 10 *S. robustum*, seven *S. spontaneum*, three *S. edule*, 11 *S.officinarium*, one *S. barberi* and one *S. sinese* were examined. The following clones important to sugarcane breeding programmes were examined, EK28, NA56-79, Nco-310, POJ2878, R570, and TUC71-7, as well as two CP clones (Canal Point, USA), two Co clones (Coimbatore, India), and four CB clones (Brazil). Clones starting with the two- or three-letter identification codes IAC (Agronomical Institute of Campinas), SP (Centro de Technologia Canaveira), and RB [Rede Interuniversitária para o Desenvolvimento do Setor Sucroalcooleiro (RIDESA)] are modern Brazilian cultivars. Nine IAC, 22 SP, and 42 RB cultivars were examined. Twenty-six modern Australian cultivars were also examined. These are the cultivars with the codes Q (Sugar Research Australia) and the older cultivars Tellus, Triton, Trojan, and Mida, clones from an old Australian breeding programme that is no longer running.

Genomic DNA was extracted from sugarcane meristems following the CTAB protocol of Aljanabi *et al.* (1999) or that of CIMMYT (2005). All samples were quantified using a NanoDrop Spectrometer (Thermo Scientific). If the absorbance reading indicated contamination, the samples were further purified using a DNeasy Plant Mini kit (Qiagen).

### Loci examined

Three loci were examined. These were named after the BAC in which the scIvana1 TE was identified. The BACs were derived from the R570 cultivar and are available in the GenBank repository [GenBank accession numbers KF184657–KF184973 at http://www.ncbi.nlm.nih.gov/genbank]. Two of the three TEs have been identified previously and classified (Domingues *et al.*, 2012): scIvana1.2 from BAC SCHRBa_044_D02 and scIvana1.1 from BAC SCHRBa_011_K15. The TE from the third BAC, SCHRBa_015_O15, was named scIvana1.4.

### qPCR

**Fig. 2.**
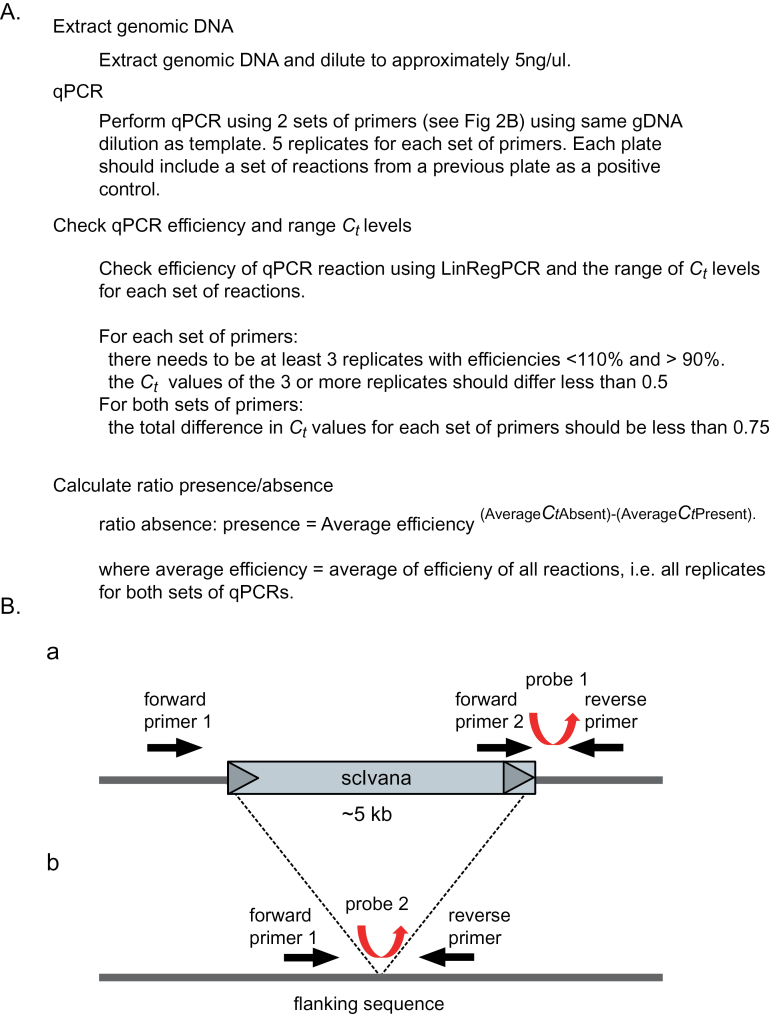
qPCR-RBIP. (A) Flow chart of the method used. (B) Schematic of the two qPCRs. Black arrows show the location of the primers, and triangles are the LTRs of the scIvana elements, depicted in grey. Flanking regions are shown as a dark grey line. Fluorescent probes used for qPCR are shown as curved arrows. When the scIvana element is present at the locus, forward primer 2 and the reverse primer are able to amplify the occupied site, while forward primer 1 and the reverse primer will not amplify because the resulting product would be too long to amplify under the PCR conditions chosen. When the scIvana element is absent at the locus, forward primer 1 and the reverse primer are able to amplify the non-occupied site because the ~5kb scIvana is not present. (This figure is available in colour at *JXB* online.)

Two sets of primers and probes were designed for each locus using the Integrated DNA Technologies website (http://www.idtdna.com/site), one set for the loci with the TE present, and one for the loci with no TE (Fig. 2). Primer and probe sequences are shown in Supplementary Table S2 (available at *JXB* online).

qPCRs were carried out in either an Applied Biosystems 7300 Real-Time System (96 -well plates) or an Applied Biosystems ABI ViiA 7 Real-Time System (384-well plates) in 15 µl reactions using the TaqMan Universal PCR Master Mix or the Taqman Gene Expression Master Mix (Applied Biosystems). The following cycling conditions were used: 50 °C for 2min, required for optimal AmpErase UNG enzyme activity, 95 °C for 10min to activate the AmpliTaq Gold DNA polymerase, followed by 40 cycles of 95 °C for 15 s and 60 °C for 1min. The genomic DNA template was at a final concentration of approximately 0.13ng µl^–1^ with five replicates for each reaction. The same actual dilution of the genomic DNA was used for each set of qPCRs, i.e. for both the presence and absence of the TE. Primer and probe concentrations were optimized according to the Applied Biosystems TaqMan Universal Master Mix manual.

The efficiency of individual PCRs was analysed using LinRegPCR (v.2014.4) (Ramakers *et al.*, 2003), which determines the slope of the exponential portion of the amplification curve of the cycle versus log fluorescence. Reactions with efficiencies of <90% or >110% were removed; if there were less than three reactions with appropriate efficiencies, the qPCR was repeated. If the *C*_t_ values for a set of three replicates differed by more than 0.5 (Nolan *et al.*, 2006), and the total of the differences in replicates for *C*_t_ values for both presence and absence was >0.75, the reactions were repeated. To test for consistency across plates, each plate included a set of reactions from a prior assay.

Average efficiency was the average efficiency for all reactions, i.e. in both the presence and absence of the TE. The ratio of the number of alleles with the TE present versus the number of alleles with the TE absent was determined by the following equation (http://www.bio-rad.com/webroot/web/pdf/lsr/literature/Bulletin_5279.pdf):

$$\begin{array}{l} \text{Ratio absence}:\text{presence}=\\ {\text{average efficiency}}^{\left(\text{Average}C\text{t absent}\right)-(\text{Average}C\text{t present})} \end{array}$$

This was then transformed into a total out of 10. For reactions where one reaction, either for the presence or absence of the scIvana element, failed to amplify for all five replicates but the converse reaction did amplify, the amplifying reaction was scored as 10. For samples where both sets of reactions failed, the genomic DNA template was tested by standard PCR with SSCIR36 microsatellite primers (Aitken *et al.*, 2005). Microsoft Excel (2013) was used to analyse the data from the ABI software and LinRegPCR (v.2014.4).

### Synteny analysis, three-dimensional (3D) scatterplots, phylogenetic analysis, and estimating the age of insertion of scIvana elements

To find regions syntenic to the three R570 BACs (SCHRBa_011_K15, SCHRBa_015_O15, and SCHRBa_044_D02), the BACs were masked and then screened against three grass genomes. The latest version of the *Sorghum bicolor* (v.2.1), *Zea mays* (v.6a), and *Oryza sativa* (v.7.0) genomes were downloaded from the JGI Phytozome v.10 site (http://phytozome.jgi.doe.gov/pz/portal.html) The masked R570 BAC sequences were queried against these genomes by BlastN from the Blast+ 2.2.29 package. Grass genomic regions were selected based on the highest score and >70% query BAC coverage. The gene and RepeatMasker TE annotation GFFs were retrieved and reformatted as GenBank files using Readseq v.2.1.30. Manual corrections were made to GenBank file tags and field names so that they were readable by Mauve. The chromosome region were submitted to the Mauve 2.3.1 (Darling *et al.*, 2010) progressive Mauve aligner and loaded into the GUI visualization Mauve program. Genes were then identified in the BACs and *S. bicolor*, *Z. mays*, and *O. sativa* and also 100kb upstream and downstream of the BACs in the syntenic regions in the three grass genomes. Genes were then queried in the Phytozome v.10 database for functional annotation, i.e. entries in the Panther, PFAM, and KEGG databases as at November 2014 (Kanehisa and Goto, 2000; Punta *et al.*, 2012; Mi *et al.*, 2013).

3D scatterplots were plotted using the package R (v.3.1.1) (http://www.r-project.org/) and the libraries rgl (v.0.94.1143) (http://cran.r-project.org/web/packages/rgl/index.html) and extrafont (v.0.16) (http://cran.r-project.org/web/packages/extrafont/index.html). A clustering dendrogram for all *S. officinarium* samples and cultivars was constructed using the package NTSYS-PC (v.2.20N) (Rohlf, 1997). A genetic distance matrix was calculated using the Nei–Li coefficient (Nei and Li, 1979). Cluster analysis based on the distance matrix was then carried out using the unweighted pair group method with arithmetic mean (UPGMA) in the SAHN subprogram of NTSYS-PC (Rohlf, 1997).

The date of insertion was calculated using 5′ and 3′ LTR divergence (Kimura two-parameter method) as implemented by MEGA5 (Tamura *et al.*, 2011), with the molecular clock equation *T=k*/2*r*, where *T* is the date of insertion, *k* is the divergence between LTR sequences, and *r* is the evolutionary rate, using the rate of 1.3 10^–8^ substitutions per site per year, as described by Ma *et al.* (2004).

## Results and discussion

Our understanding of sugarcane genetics has lagged behind that of other members of the Poaceae family such as wheat, rice, barley, and sorghum, mainly due to the hybrid nature, size, and polyploidization of the genome. Here, we used qPCR with a TE marker to create a marker system that can estimate the relative dosage of the insertion of a particular TE. We used this system to examine the evolutionary history of the *Saccharum* species complex and related genera, and showed that it can be used as a general marker, in particular, as a part of a genotyping system, or as a marker to distinguish the ancestral genomes of the modern sugarcane cultivar.

### qPCR-RBIP method

We examined the relative insertion of a TE, detected by RBIP, a PCR-based marker strategy using a three-primer set derived from the flanking region and the LTR of the LTR-RT TE (Fig. 2). We chose to develop a relative method because almost always, for a particular species or cultivar, the exact genome size and total number of alleles at a given locus is unknown. We showed that the method was reproducible across plates, machines, template concentrations, and primer concentrations. The method depends on comparing the *C*_t_ values for two sets of reactions, one for the presence of the TE and one for its absence. The following criteria were used: for each set of reactions, there were at least three replicates with efficiencies between 90 and 110% and *C*_t_ values within 0.5. In addition, the total of the differences in replicates for *C*_t_ values for both presence and absence was <0.75.

In order to compare results across plates, for each plate a set of reactions from a prior assay was re-run. Cultivars were examined both in Brazil, on an Applied Biosystems 7300 Real-Time PCR system, and in Australia, on Applied Biosystems ViiA 7 Real-Time PCR system. On the Applied Biosystems ViiA 7-Real-Time PCR system in Australia, all primers were used at a final concentrations of 500nM, while in Brazil on the 7300 Real-Time PCR system, for loci SCHRBa_044_D02 (scIvana 1.2) andSCHRBa_011_K15 (scIvana 1.1), primer concentrations were optimized at different final concentrations (Supplementary Table S2). To check that results were comparable across machines, four cultivars were tested for all three loci on both machines. For loci SCHRBa_044_D02 (scIvana 1.2) and SCHRBa_011_K15 (scIvana 1.1), they were tested at both primer concentrations. We also tested that all valid reactions, i.e. with resulting efficiencies within the parameters set, that were run on the Applied Biosystems 7300 Real-Time PCR system in Brazil using a final primer concentration of 500nM were comparable to reactions run using the optimized primer concentration. The same *C*_t_ threshold setting was used for all plates for a particular locus, i.e. the *C*_t_ threshold was not set individually for each cultivar or species. We checked that results using *C*_t_ thresholds set at 0.5, 1.0, and 1.5, within the exponential phase of the amplification plot for all cultivars, were also comparable. Finally, we also compared a 10-fold difference in genomic DNA template final concentration (Table 1 and Supplementary Fig. S1, available at *JXB* online). Estimates of the ratio of alleles with the element to alleles without the element were transformed into a total of 10. All values shown in Table 1 and Supplementary Fig. S1 are for the presence of the element, as a ratio out of 10. Differences between replicates across plates were 0.00 (min)–0.78 (max), threshold settings were 0.02–0.27, template concentrations were 0.10–0.81, and primer concentrations were 0.01–0.99. The greatest difference between replicates was across real-time PCR systems, being 0.07–1.31. A representative from Applied Biosystems suggested that this may be because the instruments have differences in their configurations, such as the optical system, light source (LED, halogen lamp), the block ramp rate, etc. (personal communication). Samples from *Miscanthus*, *Erianthus*, *S. robustum*, *S. spontaneum*, *S. edule*, most of *S. officinarium*, and all the Australian cultivars were run on the Applied Biosystems ViiA 7-Real-Time PCR system. All the Brazilian cultivars and three *S. officinarium* clones were run on the Applied Biosystems 7300 Real-Time PCR system (Supplementary Table S1). We did not normalize the results for two reasons: first, only scIvana 1.2 loci showed a higher difference compared with differences in replicates from other tests, and secondly, the greatest differences were actually found within the Brazilian cultivars (see next section).

**Table 1.** Tests of reproducibility

Test	Loci^*a*^	TE^*b*^	Number of samples	Difference between replicates^*c*^		
				Maximum	Minimum	Mean
Across plates^*d*^	SCHRBa_011_K15	scIvana 1.1	22	0.67	0.05	0.29
	SCHRBa_015_O15	scIvana 1.4	21	0.66	0.00	0.21
	SCHRBa_044_D02	scIvana 1.2	17	0.78	0.02	0.28
Across Real-Time PCR systems^*e*^	SCHRBa_011_K15	scIvana 1.1	4	0.50	0.07	0.28
	SCHRBa_015_O15	scIvana 1.4	4	0.85	0.34	0.55
	SCHRBa_044_D02	scIvana 1.2	4	1.31	0.44	0.82
Template concentration^*f*^	SCHRBa_044_D02	scIvana 1.2	8	0.81	0.10	0.37
Primer concentration^*g*^	SCHRBa_015_O15	scIvana 1.4	48	0.97	0.02	0.38
	SCHRBa_044_D02	scIvana 1.2	42	0.99	0.01	0.46
Threshold setting^*h*^	SCHRBa_011_K15	scIvana 1.1	8	0.17	0.05	0.11
	SCHRBa_015_O15	scIvana 1.4	9	0.27	0.02	0.09
	SCHRBa_044_D02	scIvana 1.2	8	0.23	0.06	0.13

^*a*^ The BAC number in which in the scIvana element was identified (de Setta *et al.*, 2014).

^*b*^ Name of the TE. scIvana 1.1 and 1.2 have been described previously (Domingues *et al.*, 2012).

^*c*^ All results are estimates of the ratio of alleles with the element to alleles without the element and were transformed into a total of 10. Only the figure for the presence of the allele is shown.

^*d*^ Master mixes were set up separately and run on two separate plates.

^*e*^ Reactions were run on either the Applied Biosystems 7300 or Applied Biosystems ViiA 7 Real-Time PCR systems.

^*f*^ Genomic DNA was added at a final concentration of 0.13 or 1.3ng μl^–1^.

^*g*^ Primers were added at a final concentration of 500nm and optimized. For optimized primer concentrations, see Supplementary Table S2.

^*h*^ Threshold settings were set at 0.5, 1.0, and 1.5.

### Using scIvana elements as markers

Modern sugarcane cultivars are aneuploid hybrids between *S. spontaneum*, a wild sugarcane, and *S. officinarum*, a traditional cultivar (Moore *et al.*, 2014). They are genetically similar, and in energy cane breeding programmes, both *S. spontaneum* and *S. robustum*, the progenitor of *S. officinarum*, are being used to broaden the genetic base and introduce traits of interest (Wang *et al.*, 2008; van der Weijde *et al.*, 2013; Santchurn *et al.*, 2014). *S. spontaneum* and two *S. robustum* were the only samples where none of the three scIvana elements were found at those genomic locations (Table 2). These are therefore the first TEs identified that could potentially be used as markers for *S. spontaneum*. Fluorescence *in situ* hybridization patterns for the sugarcane TEs, scMaximus and scDEL, do not suggest that they are specific to either *S. spontaneum* or other sugarcane species. A third element, scAle, could also potentially be used as a *S. spontaneum* specific marker, as it shows a clustering pattern and is not found on some chromosomes or chromosome arms of a modern cultivar (Domingues *et al.*, 2012). Similarly, because in *S. robustum* and *S. edule*, only scIvana1.4 is found, it could be used as a marker for these two species (Table 2). These results suggest that, combined with other markers, such as other TEs or other scIvana elements, scIvana elements could be used as markers for particular sugarcane species or groups.

**Table 2.** Proportion of alleles with the TE present in sugarcane and closely related genera

Species	No. of samples	Transposable element^*a*^/time of insertion^*b*^		
		scIvana1.4 (0.65 mya)	scIvana1.1 (0.3 mya)	scIvana1.2 (0 mya)
*Erianthus*	5	–	–	–
*Miscanthus*	5	–	–	–
*S. spontaneum*	7	0^d^	0	0
*S. robustum*	10	0.0–7.4	0	0
*S. edule*	3	2.3–8.5	0	0
*S. barberi*	1	1.4	0.4	0
*S. sinense*	1	7.9	2.9	4.4
*S. officinarium*	11	1.2–7.3	1.9–7.8	2.3–5.9
Cultivars	127	0.6–8.9	0.0–7.6	0.6–8.9

^*a*^ Name of the TE. scIvana1.1 and 1.2 have been described previously (Domingues *et al.*, 2012); scIvana1.4 was named in this paper.

^*b*^ Estimated time of insertion of TE based on 5′ and 3′ LTR divergence (Ma *et al.*, 2004). –, No amplification for presence or absence of TE; 0, amplification for absence of the TE only; other numbers or ranges indicate the minimum and maximum value for the number of alleles with the element present (as a ratio to the absence, out of 10).

Since none of the three scIvana elements were found in *S. spontaneum*, all scIvana 1.4 elements found in cultivars must have come from *S. robustum* via *S. officinarum.* The number of alleles in *S. robustum* with scIvana 1.4 present was highly variable (Fig. 1 and Table 2). Similarly, there was high variation in the number of alleles with scIavana1.2 and 1.1 present in *S. officinarum* (Fig. 1 and Table 2). In cultivars, 70–80% of the chromosomes are from *S. officinarum*, 10–23% from *S. spontaneum*, and a small portion are recombinants (D’Hont, 2005; Piperidis *et al.*, 2010). The high variation found in the cultivars is therefore probably a result of the high variation already found in *S. robustum* and *S. officinarum*. In some cultivars, there is a putative complete loss of an element; for example, in SP79-6134, there is no allele with the scIvana1.4 element present. For the *S. officinarum* samples we examined, all three elements were present at ratios ranging from 1.2 to 7.8 (Table 2). Possible reasons for the complete loss of elements in some cultivars are: (i) the parental *S. officinarum* cultivar was not included in our samples; (ii) the element was not present in the *S. officinarum* chromosomes inherited by the cultivar; and (iii) recombination with *S. spontaneum* chromosomes has resulted in the loss of the element from a chromosome.

**Fig. 3.**
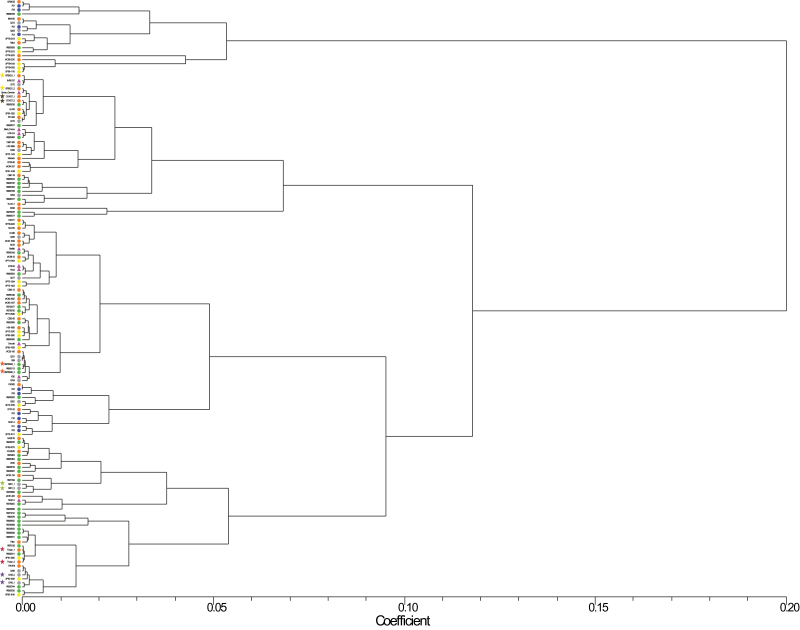
UPGMA dendogram of *S. officinarium* samples and cultivars. Coloured circles and triangles indicate the type of sample: pink triangle, *S. officinarum*; green circle, RB series from RIDESA, Brazil; yellow circle, SP series from CTC (Centro de Technologia Canaveira), Brazil; grey circle, Q canes from SRA (Sugar Research Australia), Australia; blue circle, the F series, a population from RIDESA, Brazil; orange circle, all other cultivars. Across-plate replicates are indicated by the stars.

We tested whether we could distinguish individual *S. officinarum* and cultivars. We included replicates across plates in the Nei–Li coefficient genetic distance matrix and resulting UPGMA dendrogram (indicated by stars in Fig. 3). The highest genetic distance between replicates was 0.0020 for 87S9021 (data not shown). The bootstrap values for the UPGMA were very low (<70%), so we were unable to use the UPGMA tree to distinguish the cultivars examined. These results suggested, however, that, combined with other scIvana elements or other TEs, the qPCR-RBIP method could be used to create a ‘TE profile’ of a cultivar.

### Searching for associated traits of interest

**Fig. 4.**
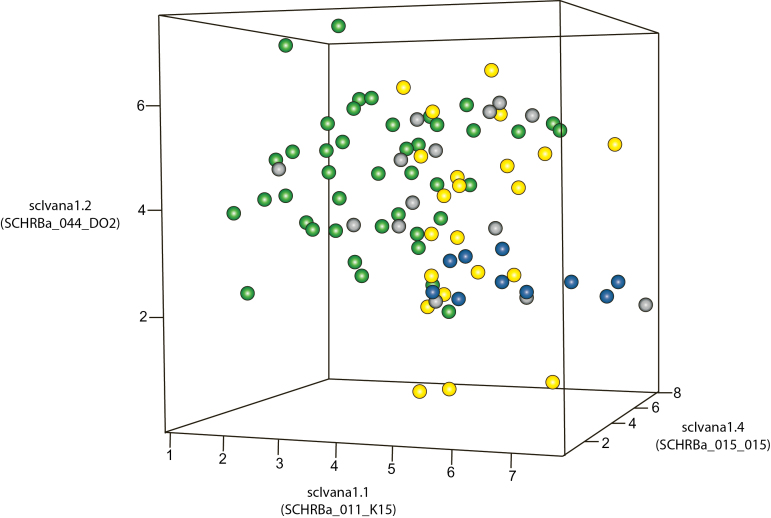
3D scatterplot for the cultivar series examined from the Australian and Brazilian breeding programmes. Green, RB series from RIDESA, Brazil; yellow, SP series from CTC (Centro de Technologia Canaveira); Brazil; grey, Q canes from SRA (Sugar Research Australia), Australia; blue, F series, a population from RIDESA, Brazil. The RB series formed a distinct cluster apart from the other cultivars.

We examined cultivars from several breeding programmes. A one- or two-letter code denotes which breeding programme the cultivar comes from. Cultivars with the two-letter code RB are from Rede Interuniversitária para o Desenvolvimento do Setor Sucroalcooleiro (RIDESA), a consortium of Brazilian Universities; those with the code F are from a single cross from RIDESA; those with the code SP are from Centro de Technologia Canaveira (CTC), the research arm of Copersucar, a Brazilian commercial company; and those with the code Q are from Sugar Research Australia (SRA). A 3D scatterplot of these cultivars showed that the RB cultivars tended to cluster apart from cultivars from other breeding programmes (Fig. 4). The Q canes and SP series were divided approximately equally between the two groups, while the F series fell into the first group away from the RB cultivars (Fig. 4). We examined whether, based on the information we had, the two groups could be distinguished by a particular trait or group of traits. In both groups, a similar percentage of cultivars fell into the main category for each trait, for example, 75% of group 1 and 73% of group 2 had average fibre content (Supplementary Table S4, available at *JXB* online). We then examined the parentage of each group. For each non-unique parent, we calculated what percentage of each group had the same parent. For group 1, every cultivar had unique parents, or shared a parent with only one other cultivar. For group 2, 44% had SP71-1088 as a parent (Supplementary Table S1). This suggested that the groupings seen in Figs 3 and 4 were the result of closely related crosses. Moreover, the genetic diversity in sugarcane breeding programmes for these loci was low.

**Fig. 5.**
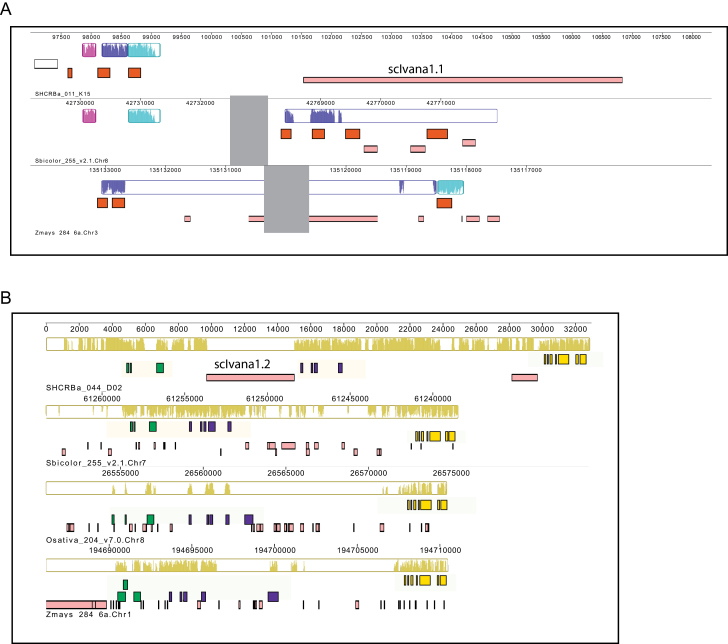
Mauve visualization of the region around the scIvana elements in sugarcane BACS and other grass genomes (*S. bicolor*, *Z. mays*, and *O. sativa*). The BAC sequence and each genome are laid out in a horizontal track. Annotated coding regions are shown as white boxes and TEs as pink boxes. A coloured similarity plot (locally collinear blocks) is shown for each genome, the height of which is proportional to the level of sequence identity in that region. The same colour represents regions of highest similarity. (A) BAC SCHRBa_011_K15 (scIvana1.1); (B) SCHRBa_044_D02 (scIvana1.2). Orange boxes, SHCRBa_011_K15.13, Sobic.008G106700, and GRMZM2G326116, translation initiation factors; green boxes, SHCRBa_044_D02.1, Sobic.007G192200, LOC_Os08g42040, and GRMZM2G083725, similar to lipid transfer protein-like; purple boxes, SHCRBa_044_D02.4, Sobic.007G192100, LOC_Os08g42050, and GRMZM2G083551), similar to coated vesicle membrane protein-like; bright pink boxes, SHCRBa_044_D02.7, Sobic.007G192000, LOC_Os08g42080, and GRMZM2G083538, ACT domain repeats.

Based on published reports of cultivar traits, we were unable to find a particular trait or groups of traits associated with the scIvana elements examined. We therefore extended our search for traits by identifying regions syntenic to and 100kb 5′ and 3′ to the BACs in three other grass genomes. *Sorghum bicolor* (v.2.1) and *Z. mays* (v.6a) are the closest fully sequenced genomes to *Saccharum*. *Oryza sativa* (v.7.0) was also chosen because it is the best annotated of the grass genomes and has been used previously in synteny analyses with sugarcane (D’Hont *et al.*, 2010; Aitken *et al.*, 2014a). Coding regions were then queried against the Phytozome v.10 database for functional annotation (Supplementary Table S3, available at *JXB* online). Syntenic regions to the BAC SCHRBa_015_O15 in the three grass genomes could not be identified with any confidence. The regions identified by the Mauve program (Darling *et al.*, 2010) contained coding regions in putatively syntenic regions to other genomes that were not listed as protein homologues in the Phytozome database. For BACs SCHRBa_011_K15 (scIvana1.1) and SCHRBa_044_D02 (scIvana1.2), a 3–4kb region around the element is illustrated in Fig. 5. Supplementary Table S3 lists in more detail the locus, location, and functional annotation from the Panther, PFAM, and KEGG databases (Kanehisa and Goto, 2000; Punta *et al.*, 2012; Mi *et al.*, 2013) for each coding region identified in the BACs and 100kb 5′ and 3′ to the BACs in the three grass genomes.

BAC SCHRBa_011_K15 was located to *S. bicolor* chromosome 8, *O. sativa* chromosome 12, and *Z. mays* chromosome 3, while SCHRBa_044_D02 was located to *S. bicolor* chromosome 7, *O. sativa* chromosome 8, and *Z. mays* chromosome 1 (Supplementary Table S3). Using the diversity arrays technology (DArTs) system, a genetic map of an Australian cultivar has been constructed in which linkage groups were clustered into eight homology groups, which represent the lowest *Saccharum* basic chromosome number (*n*=8) (Aitken *et al.*, 2014a). Good collinearity was observed between sugarcane and sorghum for four of the eight homology groups (HGs). Using this collinearity, we could localize BAC SCHRBa_011_K15 to sugarcane HG8 and BAC SCHRBa_044_D02 to sugarcane HG5.

BAC SCHRBa _044_D02 is the most gene-rich BAC (Supplementary Table S3). Probably the coding region of most interest to the domestication of the grasses is the coding region Sobic.007G193500 (*S. bicolor* chromosome 7, 6137404–6133899). *SQUAMOSA-PROMOTER BINDING PROTEIN-LIKE* (*SPL*) proteins are a diverse family of transcription factors that play fundamental roles in plant growth and development (Preston and Hileman, 2013). The most extensively characterized SPL gene in maize is the *teosinte glume architecture 1* (*tga1*) gene. The key event in the domestication of *Z. mays* was the liberation of the kernel in the maize progenitor, teosinte (Dorweiler *et al.*, 1993). This event is controlled by variation in the *tag1* gene (Wang *et al.*, 2005).

### Evolutionary history of scIvana in sugarcane

The RBIP method is based on the insertion of LTR-RTs, which are usually irreversible. In addition, it is possible to date the time of insertion of an element. These two features mean that it was possible to trace the history of insertion and timing of the three scIvana elements. For all 10 *Miscanthus* and *Erianthus* species tested, there was no amplicon for either reaction. Templates tested by standard PCR with SSCIR36 microsatellite primers (Aitken *et al.*, 2005) resulted in amplicons of the expected size (data not shown). This suggested that the loci are not present in *Miscanthus* or *Erianthus*. The age of insertion of the scIvana elements was consistent with them being found only in the *Saccharum* lineage. The nucleotide divergence of the LTRs of LTR-RTs can be used to date the time of insertion of the element. The date of insertion was calculated using the nucleotide divergence between the 5′ and 3′ LTRs with a rate of 1.3×10^–8^ substitutions per site per year, as described by Ma *et al.* (2004). The time of insertion for the scIvana elements 1.1 and 1.4 were estimated at 0.30 and 0.65 million years ago (mya), respectively. The LTRs of scIvana 1.2 are 100% identical, and the estimated time of divergence is therefore 0 mya. The time of divergence between *Saccharum* and *Erianthus*/*Miscanthus* is estimated to be ~3.8–4.6 mya (Kim *et al.*, 2014).

In *S. spontaneum*, a wild *Saccharum* species, qPCR-RBIP for the absence of the all three elements resulted in an amplicon, while the reaction for the presence of all three elements resulted in no amplification. This suggested that all three loci were present, but that there were no scIvana elements present at any allele (Fig. 1, Table 2). The first element to appear in *Saccharum* was scIvana1.4, in *S. robustum*, the wild *Saccharum* species used in the breeding of the modern cultivars (Fig. 1, Table 2). This element is also found in *S. edule*, thought to be a mutant of *S. robustum* clones (Grivet *et al.*, 2006). Ten *S. robustum* and three *S.edule* clones were examined. Values for the ratio of the presence of scIavana 1.4 varied widely (0–7.4 for *S. robustum* and 2.3–8.5 for *S. edule*). For two *S. robustum* clones, IJ76-507 and IM76-229, no scIvana 1.4 element was detected.

The other two scIvana elements, 1.2 and 1.1, arose in the lineage leading to *S. officinarum* (Fig. 1, Table 2), the traditional cultivar used in the breeding of the modern cultivars. The older traditional cultivars, *S. barberi* and *S. sinense*, are thought to be natural hybrids between *S. officinarum* and *S. robustum* (D’Hont *et al.*, 2002). Our data were limited for *S. barberi* and *S. sinense*, as we examined only a single clone of each. All three scIvana elements were identified in *S. sinense*, while scIvana 1.1 and 1.4 were found in *S. barberi*. ScIvana 1.1 and 1.4 had older insertion times compared with scIvana 1.2 (0 mya). All three scIvana elements were also found in all *S. officinarum* clones and all cultivars except for scIvana 1.1 in EK28 and scIvana 1.2 in cultivars CP74-2005, SP79-6134, SP79-6192, and SP89-1115.

The pattern and timing of insertion of the elements supports the current scenario for the evolution of sugarcane (Grivet *et al.*, 2006). This example demonstrates how the RBIP method can be used to determine the evolutionary history of individual LTR-RTs or the timing of divergence of taxa, based on the timing of the appearance of elements in a taxon.

## Conclusions

Based on genome sequence and LTR-RT identification, we established a method using qPCR with the RBIP marker system (qPCR-RBIP) to estimate the ratio of alleles at a particular locus with a scIvana TE. Using a criteria of three replicates with efficiencies between 90 and 110% and a total of the differences in replicates for *C*_t_ values for both presence and absence of <0.75, we were able to establish a system that is reproducible across the main sources of error, for example across plates, template concentrations, and threshold levels. All three elements screened, scIvana1.1, 1.2, and 1.4, are specific to the *S. robustum–S. officinarum* modern cultivars lineage and are therefore the first TEs identified that could potentially be used as markers for *S. spontaneum.* Within the *S. officinarum* modern cultivars group, in which all three elements were found, the qPCR-RBIP method has the potential to be used to produce an individual ‘TE profile’ of a cultivar. Finally, we demonstrated how the RBIP system can be used to trace the timing of divergence of taxa within *Saccharum* species.

## Supplementary data

Supplementary data are available at *JXB* online.

Supplementary Table S1. Samples examined.

Supplementary Table S2. Sequences and concentrations of primers and probes used.

Supplementary Table S3. Genomic neighbourhood of scIvana elements.

Supplementary Table S4. For each group identified by phylogenetic analysis (Supplementary Fig. S2), the percentage of cultivars from the Australian and Brazilian breeding programmes (RB, SP, Q canes and the F series) that fall into each trait scale.

Supplementary Fig. S1. Tests of reproducibility.

Supplementary Fig. S2. UPGMA dendogram of the cultivar series examined from Australia and Brazilian breeding programmes.

Supplementary Data

## Abbreviations:

BAC: bacterial artificial chromosome
*C*_*t*_: cycle threshold
HG: homology group
LTR-RT: long terminal repeat retrotransposon
mya: million years ago
qPCR: quantitative PCR
RBIP: retrotransposon-based insertion polymorphism
RIDESA: Rede Interuniversitária para o Desenvolvimento do Setor Sucroalcooleiro
TE: transposable element
TSD: target site duplication
UPGMA: unweighted pair group method with arithmetic mean.

## References

[CIT0001] Aitken KS Jackson PA McIntyre CL 2005 A combination of AFLP and SSR markers provides extensive map coverage and identification of homo(eo)logous linkage groups in a sugarcane cultivar. Theoretical and Applied Genetics 110, 789–801. 15700149 10.1007/s00122-004-1813-7

[CIT0002] Aitken KS Li J-C Jackson P Piperidis G McIntyre CL 2006 AFLP analysis of genetic diversity within *Saccharum officinarum* and comparison with sugarcane cultivars. Australian Journal of Agricultural Research 57, 1167–1184.

[CIT0003] Aitken KS McNeil MD Berkman PJ Hermann S Kilian A Bundock PC Li J 2014 *a* Comparative mapping in the Poaceae family reveals translocations in the complex polyploid genome of sugarcane. BMC Plant Biology 14, 190. 25059596 10.1186/s12870-014-0190-x PMC4222257

[CIT0004] Aitken KS McNeil MD Hermann S Bundock PC Kilian A Heller-Uszynska K Henry RJ Li J 2014 *b* A comprehensive genetic map of sugarcane that provides enhanced map coverage and integrates high-throughput Diversity Array Technology (DArT) markers. BMC Genomics 15, 152. 24564784 10.1186/1471-2164-15-152 PMC4007999

[CIT0005] Alexander A 1985 The energy cane alternative. Amsterdam, The Netherlands: Elsevier.

[CIT0006] Aljanabi S Forget L Dookun A 1999 An improved and rapid protocol for the isolation of polysaccharide-and polyphenol-free sugarcane DNA. Plant Molecular Biology Reporter 17, 1–8.

[CIT0007] Alwala S Suman A Arro JA Veremis JC Kimbeng CA 2006 Target region amplification polymorphism (TRAP) for assessing genetic diversity in sugarcane germplasm collections. Crop Science 46, 448–455.

[CIT0008] Arro J Veremis J Kimbeng C Botanga C 2006 Genetic diversity and relationships revealed by AFLP markers among *Saccharum spontaneum* and related species and genera. Journal of the American Society Sugar Cane Technologists 26, 101–115.

[CIT0009] Berkman PJ Bundock PC Casu RE Henry RJ Rae AL Aitken KS 2014 A survey sequence comparison of *Saccharum* genotypes reveals allelic diversity differences. Tropical Plant Biology 7, 71–83.

[CIT0010] Botha FC Moore PH 2014 Biomass and bio-energy. In: Moore PH Botha FC, eds. Sugarcane: physiology, biochemistry, and functional biology. Ames, IA: John Wiley & Sons, 521–540.

[CIT0011] Bremer G 1961 Problems in breeding and cytology of sugar cane. III. The cytological crossing research of sugar cane. Euphytica 10, 229–243.

[CIT0012] Cardoso-Silva CB Costa EA Mancini MC Balsalobre TWA Canesin LEC Pinto LR Carneiro MS Garcia AAF de Souza AP Vicentini R 2014 De novo assembly and transcriptome analysis of contrasting sugarcane varieties. PLOS ONE 9, e88462. 24523899 10.1371/journal.pone.0088462 PMC3921171

[CIT0013] Chong BF O’Shea MG 2012 Developing sugarcane lignocellulosic biorefineries: opportunities and challenges. Biofuels 3, 307–319.

[CIT0014] **CIMMYT**. 2005 *Laboratory protocols: CIMMYT Applied Molecular Genetics Laboratory*. Mexico: CIMMYT.

[CIT0015] Cordeiro GM Pan YB Henry RJ 2003 Sugarcane microsatellites for the assessment of genetic diversity in sugarcane germplasm. Plant Science 165, 181–189.

[CIT0016] D’Hont A Garsmeur O McIntyre L 2010 Mapping, tagging and map-based cloning of simply inherited traits. In: Henry R Kole C, eds. Genetics, genomics and breeding of sugarcane. Enfield, USA: Science Publishers, 97–115.

[CIT0017] D’Hont A Glaszmann JC 2001 Sugarcane genome analysis with molecular markers: a first decade of research. In: International Society of Sugar Cane Technologists. Proceedings of the XXIV Congress, 556–559.

[CIT0018] D’Hont A Ison D Alix K Roux C Glaszmann JC 1998 Determination of basic chromosome numbers in the genus *Saccharum* by physical mapping of ribosomal RNA genes. Genome 41, 221–225.

[CIT0019] D’Hont A Paulet F Glaszmann JC 2002 Oligoclonal interspecific origin of ‘North Indian’ and ‘Chinese’ sugarcanes. Chromosome Research 10, 253–262. 12067214 10.1023/a:1015204424287

[CIT0020] D’Hont A 2005 Unraveling the genome structure of polyploids using FISH and GISH; examples of sugarcane and banana. Cytogenetic and Genome Research 109, 27–33. 15753555 10.1159/000082378

[CIT0021] Dal-Bianco M Carneiro MS Hotta CT Chapola RG Hoffmann HP Garcia AAF Souza GM 2012 Sugarcane improvement: how far can we go? Current Opinion in Biotechnology 23, 265–70. 21983270 10.1016/j.copbio.2011.09.002

[CIT0022] Darling AE Mau B Perna NT 2010 progressiveMauve: multiple genome alignment with gene gain, loss and rearrangement. PLOS ONE 5, e11147. 20593022 10.1371/journal.pone.0011147 PMC2892488

[CIT0023] de Setta N Monteiro-Vitorello CB Metcalfe CJ 2014 Building the sugarcane genome for biotechnology and identifying evolutionary trends. BMC Genomics 15, 540. 24984568 10.1186/1471-2164-15-540 PMC4122759

[CIT0024] Devarumath RM Kalwade SB Bundock P Eliott FG Henry R 2013 Independent target region amplification polymorphism and single-nucleotide polymorphism marker utility in genetic evaluation of sugarcane genotypes. Plant Breeding 132, 736–747.

[CIT0025] Domingues D 2009 Sure e Garapa: Caracterização Molecular e Distribuição de Dois Retrotransposons com LTR de Cana-de-Açúcar. PhD thesis, University of São Paulo.

[CIT0026] Domingues DS Cruz GMQ Metcalfe CJ Nogueira FTS Vicentini R de S Alves C Van Sluys M-A 2012 Analysis of plant LTR-retrotransposons at the fine-scale family level reveals individual molecular patterns. BMC Genomics 13, 137. 22507400 10.1186/1471-2164-13-137 PMC3352295

[CIT0027] Dorweiler J Stec A Kermicle J Doebley J 1993 *Teosinte glume architecture 1*: a genetic locus controlling a key step in maize evolution. Science 262, 233–235. 17841871 10.1126/science.262.5131.233

[CIT0028] Garcia AAF Mollinari M Marconi TG 2013 SNP genotyping allows an in-depth characterisation of the genome of sugarcane and other complex autopolyploids. Scientific Reports 3, 3399. 24292365 10.1038/srep03399 PMC3844970

[CIT0029] Grativol C Regulski M Bertalan M 2014 Sugarcane genome sequencing by methylation filtration provides tools for genomic research in the genus *Saccharum* . The Plant Journal 79, 162–172. 24773339 10.1111/tpj.12539 PMC4458261

[CIT0030] Grivet L Glaszmann J D’Hont A 2006 Molecular evidence of sugarcane evolution and domestication. In: Motley T Nyree Z Cross H, eds. Darwin’s harvest, new approaches to the origins, evolution and conservation of crops. New York: Columbia University Press, 49–66.

[CIT0031] Henry R Kole C 2010 Genetics, genomics and breeding of sugarcane. Enfield, USA: Science Publishers.

[CIT0032] Hill J Nelson E Tilman D Polasky S Tiffany D 2006 Environmental, economic, and energetic costs and benefits of biodiesel and ethanol biofuels. Proceedings of the National Academy of Sciences, USA 103, 11206–11210. 10.1073/pnas.0604600103 PMC1544066 16837571

[CIT0033] Hotta CT Lembke CG Domingues DS 2010 The biotechnology roadmap for sugarcane improvement. Tropical Plant Biology 3, 75–87.

[CIT0034] Kanehisa M Goto S 2000 KEGG: Kyoto encyclopedia of genes and genomes. Nucleic Acids Research 28, 27–30. 10592173 10.1093/nar/28.1.27 PMC102409

[CIT0035] Kim C Wang X Lee T-H Jakob K Lee G-J Paterson AH 2014 Comparative analysis of *Miscanthus* and *Saccharum* reveals a shared whole-genome duplication but different evolutionary fates. The Plant Cell 26, 2420–2429. 24963058 10.1105/tpc.114.125583 PMC4114942

[CIT0036] Kumar A Hirochika H 2001 Applications of retrotransposons as genetic tools in plant biology. Trends in Plant Science 6, 127–134. 11239612 10.1016/s1360-1385(00)01860-4

[CIT0037] Lu YH D’Hont A Walker DIT Rao PS Feldmann P Glaszmann JC 1994 Relationships among ancestral species of sugarcane revealed with RFLP using single copy maize nuclear probes. Euphytica 78, 7–18.

[CIT0038] Ma J Devos KM Bennetzen JL 2004 Analyses of LTR-retrotransposon structures reveal recent and rapid genomic DNA loss in rice. Genome Research 14, 860–869. 15078861 10.1101/gr.1466204 PMC479113

[CIT0039] Matsuoka S Kennedy AJ Santos EGD dos Tomazela AL Rubio LCS 2014 Energy cane: its concept, development, characteristics, and prospects. Advances in Botany 2014, 1–13.

[CIT0040] Mi H Muruganujan A Thomas PD 2013 PANTHER in 2013: modeling the evolution of gene function, and other gene attributes, in the context of phylogenetic trees. Nucleic Acids Research 41, D377–D386. 23193289 10.1093/nar/gks1118 PMC3531194

[CIT0041] Ming R Del Monte TA Hernandez E Moore PH Irvine JE Paterson AH 2002 Comparative analysis of QTLs affecting plant height and flowering among closely-related diploid and polyploid genomes. Genome 45, 794–803. 12416611 10.1139/g02-042

[CIT0042] Ming R Liu SC Moore PH Irvine JE Paterson AH 2001 QTL analysis in a complex autopolyploid: genetic control of sugar content in sugarcane. Genome Research 11, 2075–84. 11731498 10.1101/gr.198801 PMC311218

[CIT0043] Moore PH Paterson AH Tew T 2014 Sugarcane: the crop, the plant, and domestication. Moore PH Botha FC, eds. Ames, Iowa, USA: John Wiley & Sons.

[CIT0044] Nair NV Nair S Sreenivasan T V Mohan M 1999 Analysis of genetic diversity and phylogeny in *Saccharum* and related genera using RAPD markers. Genetic Resources and Crop Evolution 46, 73–79.

[CIT0045] Nei M Li WH 1979 Mathematical model for studying genetic variation in terms of restriction endonucleases. Proceedings of the National Academy of Sciences, USA 76, 5269–5273. 10.1073/pnas.76.10.5269 PMC413122 291943

[CIT0046] Nishiyama MY Ferreira SS Tang P-Z Becker S Pörtner-Taliana A Souza GM 2014 Full-length enriched cDNA libraries and ORFeome analysis of sugarcane hybrid and ancestor genotypes. PLOS ONE 9, e107351. 25222706 10.1371/journal.pone.0107351 PMC4164538

[CIT0047] Nolan T Hands RE Bustin SA 2006 Quantification of mRNA using real-time RT-PCR. Nature Protocols 1, 1559–1582. 10.1038/nprot.2006.236 17406449

[CIT0048] Pan YB Scheffler BE Richard E 2007 High-throughput molecular genotyping of commercial sugarcane clones with microsatellite (SSR) markers. Sugar Tech 9, 176–181.

[CIT0049] Parida SK Kalia SK Kaul S 2009 Informative genomic microsatellite markers for efficient genotyping applications in sugarcane. Theoretical and Applied Genetics 118, 327–338. 18946655 10.1007/s00122-008-0902-4

[CIT0050] Pastina MM Pinto LR Oliveira KM de Souza AP Garcia AAF 2010 molecular mapping of complex traits. In: Henry RJ Kole C, eds. Genetics, genomics and breeding of sugarcane. Enfield, USA: Science Publishers, 117–147.

[CIT0051] Pinto LR Oliveira KM Ulian EC Garcia AAF de Souza AP 2004 Survey in the sugarcane expressed sequence tag database (SUCEST) for simple sequence repeats. Genome 47, 795–804. 15499394 10.1139/g04-055

[CIT0052] Piperidis G Piperidis N D’Hont A 2010 Molecular cytogenetic investigation of chromosome composition and transmission in sugarcane. Molecular Genetics and Genomics 284, 65–73. 20532565 10.1007/s00438-010-0546-3

[CIT0053] Preston JC Hileman LC 2013 Functional evolution in the plant *SQUAMOSA-PROMOTER BINDING PROTEIN-LIKE* (*SPL*) gene family. Frontiers in Plant Science 4, 80. 23577017 10.3389/fpls.2013.00080 PMC3617394

[CIT0054] Punta M Coggill PC Eberhardt RY 2012 The Pfam protein families database. Nucleic Acids Research 40, D290–D301. 22127870 10.1093/nar/gkr1065 PMC3245129

[CIT0055] Ramakers C Ruijter JM Deprez RHL Moorman AFM 2003 Assumption-free analysis of quantitative real-time polymerase chain reaction (PCR) data. Neuroscience Letters 339, 62–66. 12618301 10.1016/s0304-3940(02)01423-4

[CIT0056] Ritter E Gebhardt C Salamini F 1990 Estimation of recombination frequencies and construction of RFLP linkage maps in plants from crosses between heterozygous parents. Genetics 125, 645–654. 1974227 10.1093/genetics/125.3.645 PMC1204090

[CIT0057] Roach B 1972 Nobilisation of sugarcane. Proceedings of the International Society of Sugar Cane Technologists 14, 206–216.

[CIT0058] Rohlf F 1997 NTSYS-PC: Numerical taxonomy and multivariate analysis system. Version 2.1 Applied Biostatistics.

[CIT0059] Santchurn D Ramdoyal K Badaloo MGH Labuschagne MT 2014 From sugar industry to cane industry: evaluation and simultaneous selection of different types of high biomass canes. Biomass and Bioenergy 61, 82–92.

[CIT0060] Sims REH Mabee W Saddler JN Taylor M 2010 An overview of second generation biofuel technologies. Bioresource Technology 101, 1570–1580. 19963372 10.1016/j.biortech.2009.11.046

[CIT0061] Tamura K Peterson D Peterson N Stecher G Nei M Kumar S 2011 MEGA5: molecular evolutionary genetics analysis using maximum likelihood, evolutionary distance, and maximum parsimony methods. Molecular Biology and Evolution 28, 2731–2739. 21546353 10.1093/molbev/msr121 PMC3203626

[CIT0062] van der Weijde T Alvim Kamei CL Torres AF Vermerris W Dolstra O Visser RGF Trindade LM 2013 The potential of C4 grasses for cellulosic biofuel production. Frontiers in Plant Science 4, 107. 23653628 10.3389/fpls.2013.00107 PMC3642498

[CIT0063] Waclawovsky AJ Sato PM Lembke CG Moore PH Souza GM 2010 Sugarcane for bioenergy production: an assessment of yield and regulation of sucrose content. Plant Biotechnology Journal 8, 263–276. 20388126 10.1111/j.1467-7652.2009.00491.x

[CIT0064] Wang H Nussbaum-Wagler T Li B Zhao Q Vigouroux Y Faller M Bomblies K Lukens L Doebley JF 2005 The origin of the naked grains of maize. Nature 436, 714–719. 16079849 10.1038/nature03863 PMC1464477

[CIT0065] Wang L-P Jackson PA Lu X Fan Y-H Foreman JW Chen X-K Deng H-H Fu C Ma L Aitken KS 2008 Evaluation of sugarcane*×Saccharum spontaneum* progeny for biomass composition and yield components. Crop Science 48, 951–961.

[CIT0066] Wicker T Sabot F Hua-Van A 2007 A unified classification system for eukaryotic transposable elements. Nature Reviews Genetics 8, 973–982. 10.1038/nrg2165 17984973

[CIT0067] Wu KK Burnquist W Sorrells ME Tew TL Moore PH Tanksley SD 1992 The detection and estimation of linkage in polyploids using single-dose restriction fragments. Theoretical and Applied Genetics 83, 294–300. 24202510 10.1007/BF00224274

[CIT0068] Zhang J Zhou M Walsh J Zhu L Chen Y Ming R 2014 Sugarcane genetics and genomics. In: Moore PH Botha FC, eds. Sugarcane: physiology, biochemistry, and functional biology. Ames, IA: John Wiley & Sons, 623–643.

[CIT0069] Zinoviev S Müller-Langer F Das P Bertero N Fornasiero P Kaltschmitt M Centi G Miertus S 2010 Next-generation biofuels: survey of emerging technologies and sustainability issues. ChemSusChem 3, 1106–1133. 20922754 10.1002/cssc.201000052

