# Supplementary material for: Using quantitative PCR with retrotransposon-based insertion polymorphisms as markers in sugarcane
Source: J Exp Bot. 2015 Jun 19;66(14):4239–50. doi: 10.1093/jxb/erv283 (PMC4493790; doi:10.1093/jxb/erv283)
Supplement: Supplementary Data [file supp_66_14_4239__index.html]

Using quantitative PCR with retrotransposon-based insertion polymorphisms as markers in sugarcane — Using quantitative PCR with retrotransposon-based insertion polymorphisms as markers in sugarcane — Supplementary Data 

# Using quantitative PCR with retrotransposon-based insertion polymorphisms as markers in sugarcane

## Supplementary Data

Data files

- Supplementary Data - Supplementary Data
- Supplementary Data - Supplementary Data
- Supplementary Data - Supplementary Data
