# Supplementary material for: Using quantitative PCR with retrotransposon-based insertion polymorphisms as markers in sugarcane
Source: J Exp Bot. 2015 Jun 19;66(14):4239–50. doi: 10.1093/jxb/erv283 (PMC4493790; doi:10.1093/jxb/erv283)
Supplement: Supplementary Data [file supp_erv283_jexbot151100_file001.pdf]

Journal of Experimental Botany

Using quantitative PCR with retrotransposon-insertion polymorphisms as markers in sugarcane

Cushla J. Metcalfe, Sarah G. Oliveira, Jonas W. Gaiarsa, Karen S. Aitken, Monalisa S. Carneiro, Fernanda Zatti, Marie-Anne Van Sluys

Supplementary Figure 1. Tests of Reproducibility. Bars are the number of alleles with the element present as a ratio out of 10. Error bars are the total difference in Ct values for all replicates (i.e. for all reactions analysed, for both the presence and the absence of the element). Figures show replicates across A. plates, B. Real-Time PCR Systems, C. template concentration, D. primer concentration and E. threshold settings.

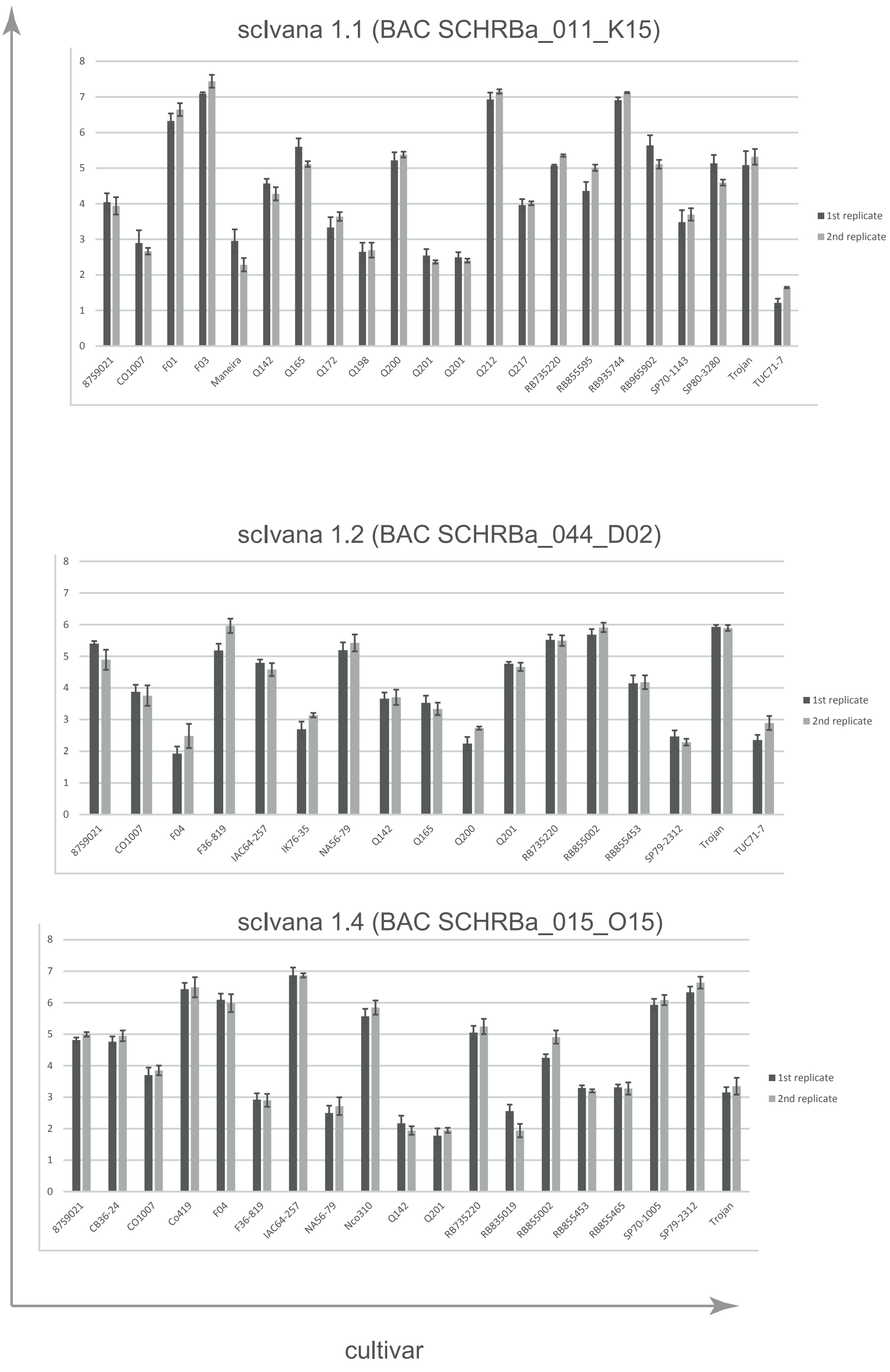

Supplementary Figure S1.A

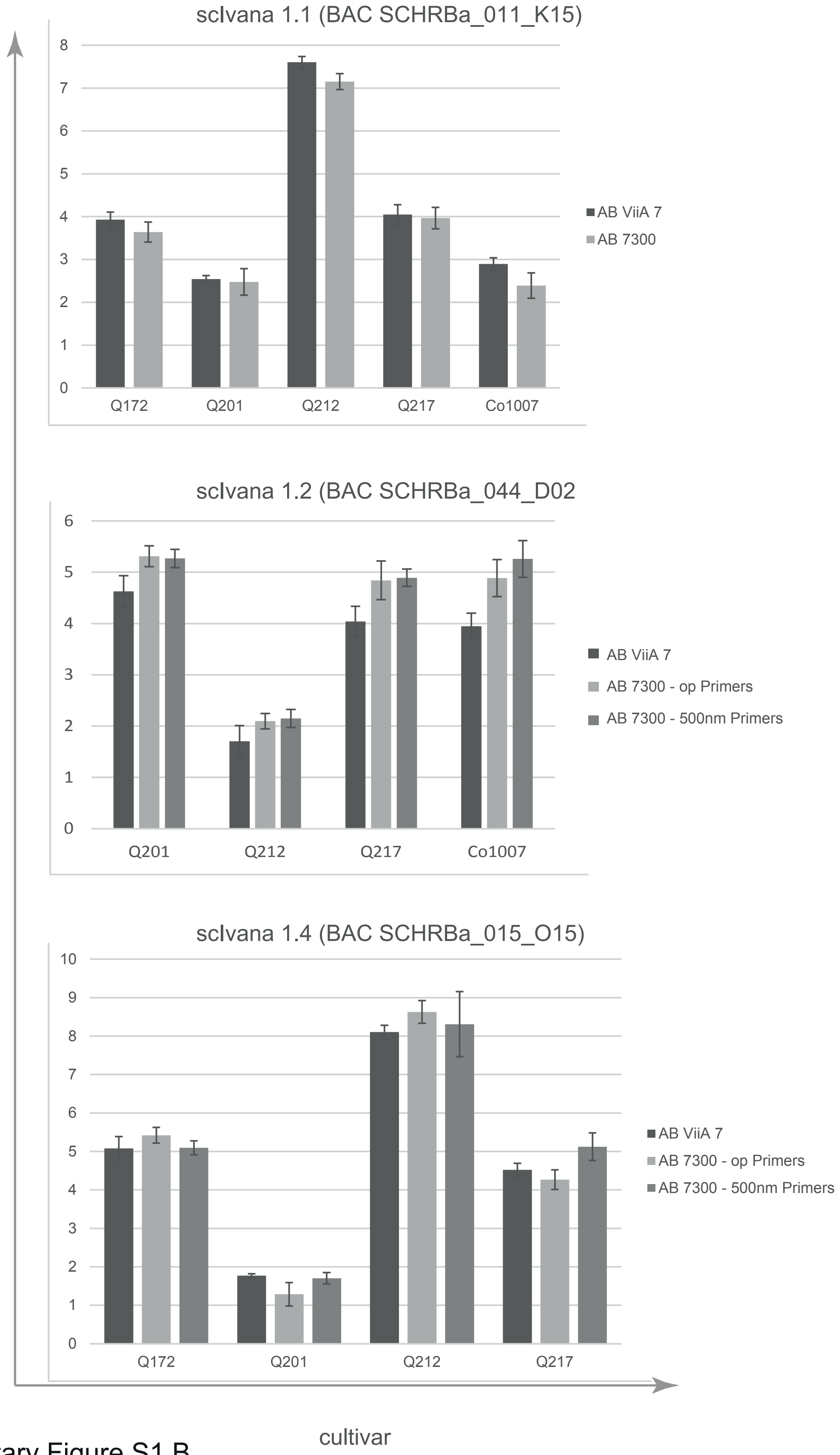

Supplementary Figure S1.B

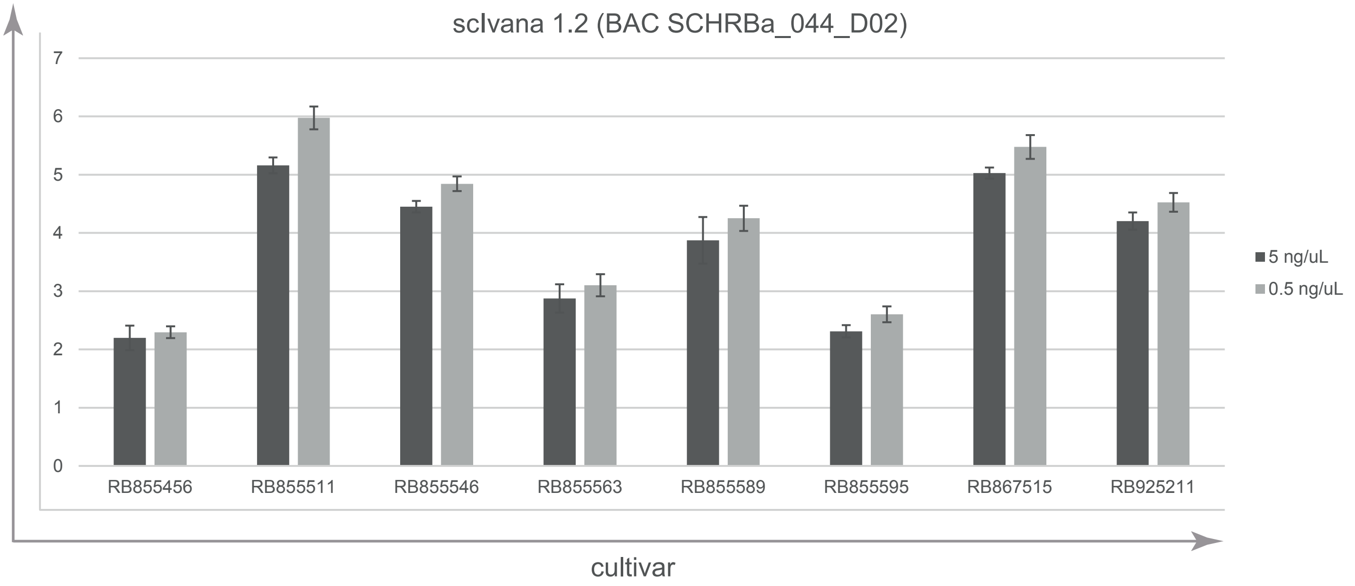

Supplementary Figure S1.C

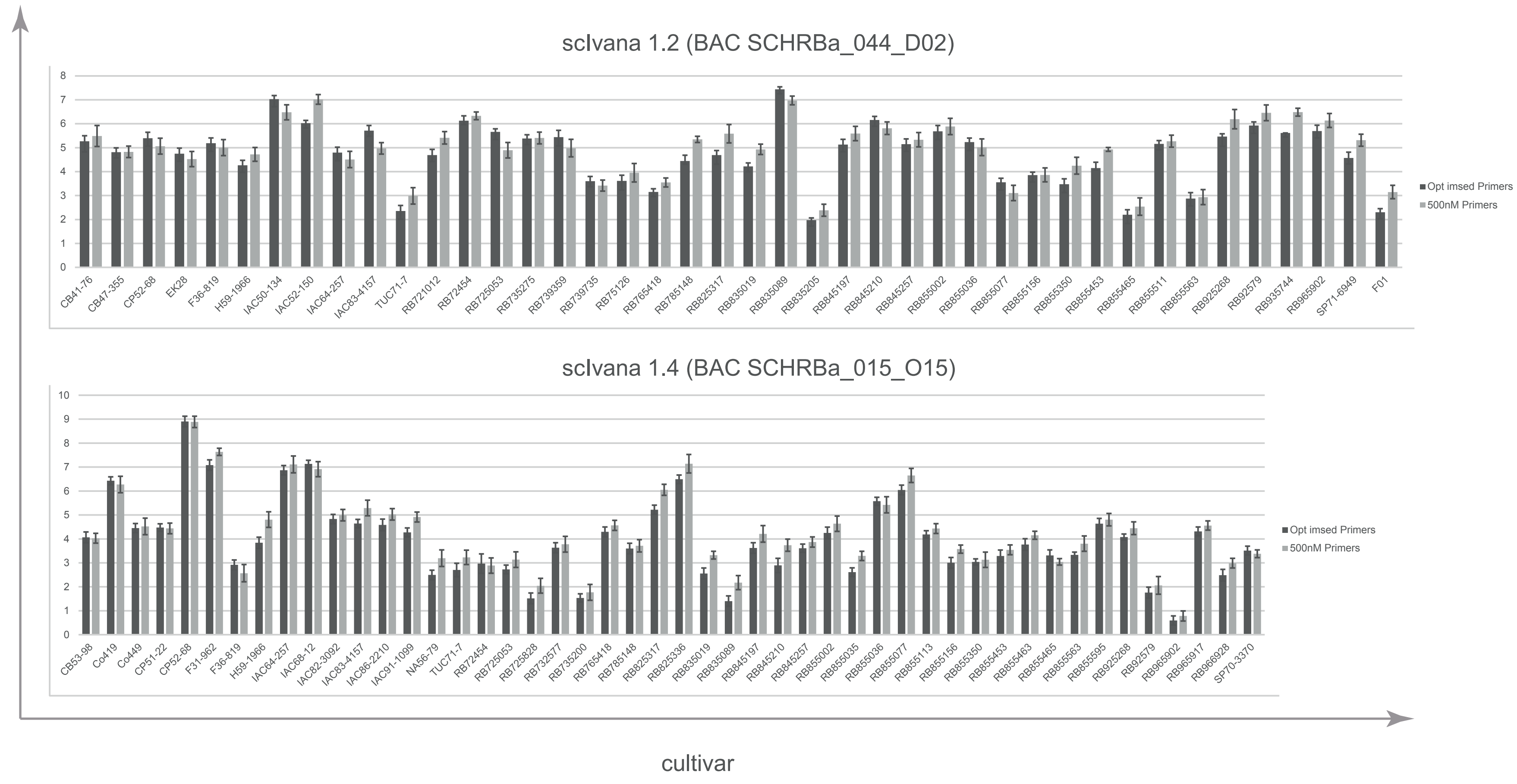

Supplementary Figure S1.D

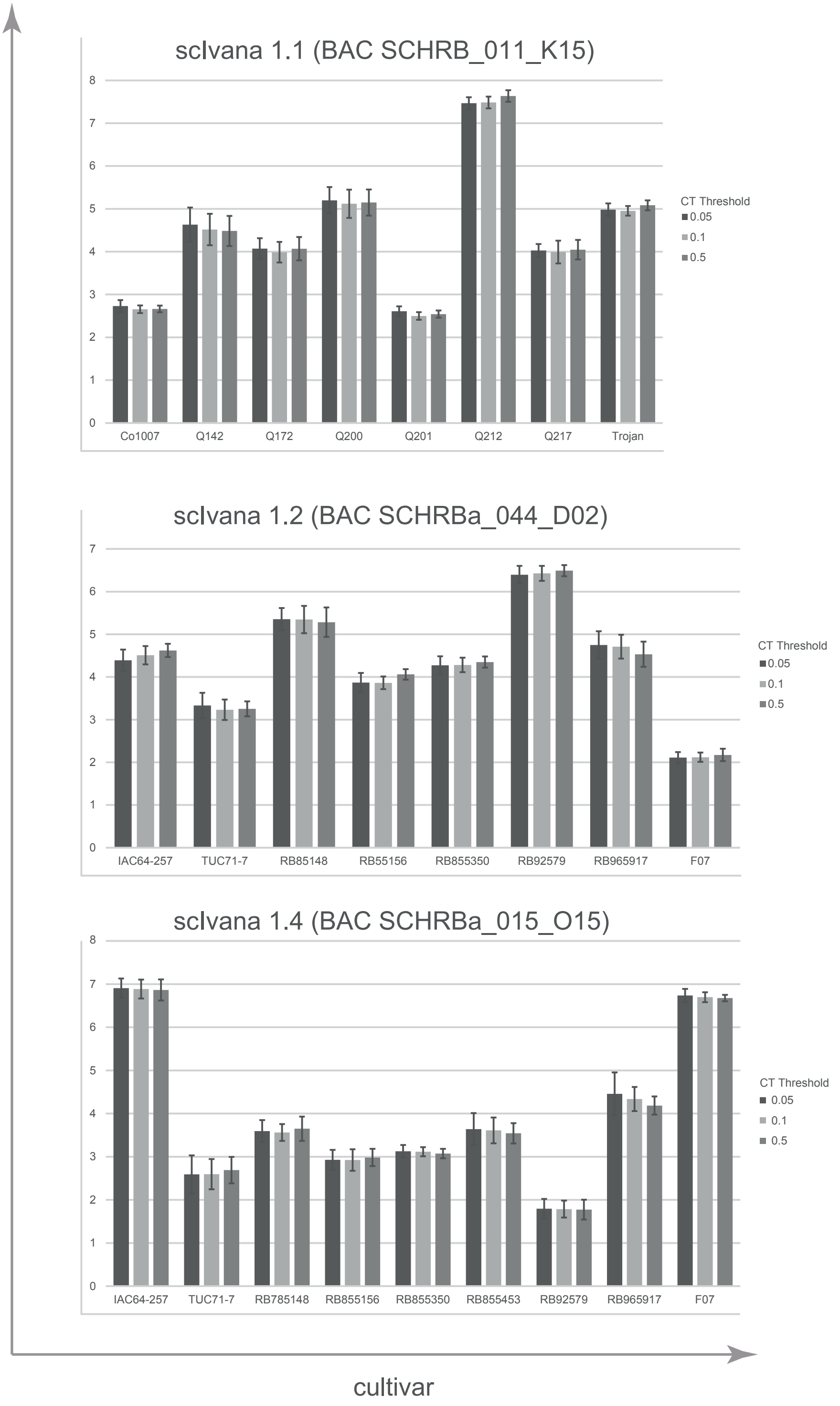

Supplementary Figure S1.E

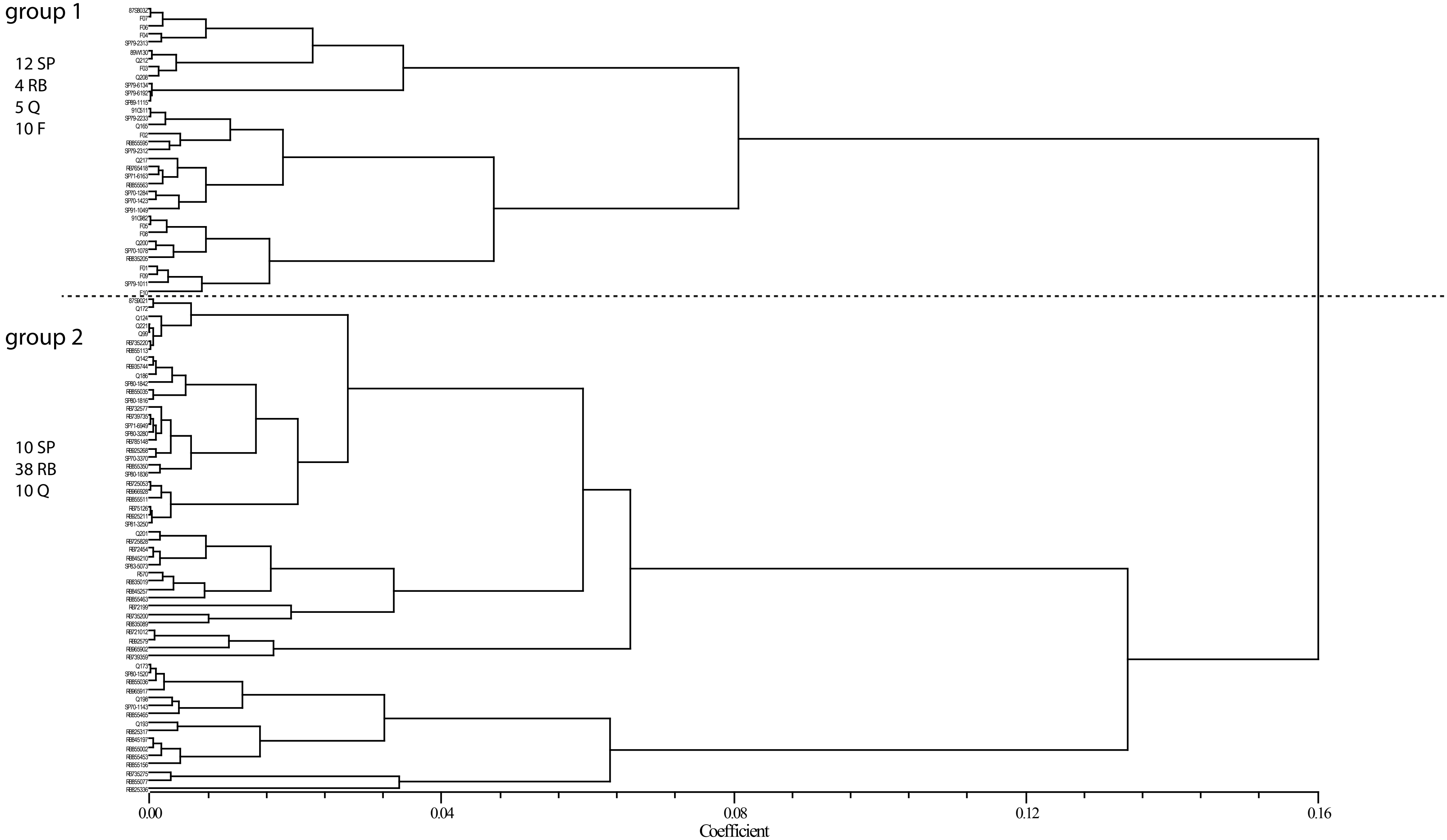

Supplementary Figure S2. UPGMA dendogram of the cultivar series examined from Australia and Brazilian breeding programs; the RB series from RIDESA, Brazil; the SP series from CTC (Centro de Technologia Canaveira), Brazil; Q-canes from SRA (Sugar Research Australia), Australia and the F series, a population from RIDESA, Brazil. The dotted line indicates the division between chiefly RB cultivars and all other cultivars.

Genus/ species	cultivar/ accession	Parent 1	Parent 2	Loci 11K15	Loci 15015	Loci 44D02	Real-Time PCR system used	Provenance	Sucrose content	Fibre content	Smut	Brown rust	Leaf scald	Sugarcane Mosaci Virus (SGMV)
Cultivar	87S803Z			3.48	6.16	1.80	ViiA	Australia						
Cultivar	87S9021			4.04	4.81	5.40	ViiA	Australia						
Cultivar	89W130			7.64	7.86	1.84	ViiA	Australia						
Cultivar	91C511			5.00	4.51	2.87	ViiA	Australia						
Cultivar	91C982			5.27	2.89	2.80	ViiA	Australia						
Cultivar	CB40-13	POJ2878	Co290	4.96	3.88	4.74	700	Brazil						
Cultivar	CB41-76	POJ2878	?	2.52	4.17	5.27	700	Brazil	3					
Cultivar	CB47-355	POJ2878	Co413	3.23	7.10	4.81	700	Brazil	2					
Cultivar	CB53-98	CB46-40	?	6.28	4.07	5.12	700	Brazil	3					
Cultivar	CO1007			2.90	3.70	3.88	ViiA	Coimbatore, India						
Cultivar	Co419	POJ2878	Co290	4.25	6.43	5.62	700	India	3	4	8		4	
Cultivar	Co449	POJ2878	Co331	5.53	4.45	3.23	700	India			1			
Cultivar	CP51-22	F36-819	CP33-372	5.46	4.47	2.21	700	USA	3					
Cultivar	CP52-68	CP29-320		5.33	8.90	5.40	700	USA						
Cultivar	CP74-2005	EK2	POJ100	1.61	3.96	0.00	ViiA	Canal Point, USA						
Cultivar	EK28	EK2	POJ100	0.00	5.83	4.75	700							
Cultivar	F01	SP80-3280	RB835486	6.32	4.08	2.31	700	Brazil						
Cultivar	F02	SP80-3280	RB835486	5.56	5.19	2.41	700	Brazil						
Cultivar	F03	SP80-3280	RB835486	7.09	7.97	2.24	700	Brazil						
Cultivar	F04	SP80-3280	RB835486	4.42	6.09	1.93	700	Brazil						
Cultivar	F05	SP80-3280	RB835486	6.06	3.36	3.18	700	Brazil						
Cultivar	F06	SP80-3280	RB835486	4.10	7.60	2.78	700	Brazil						
Cultivar	F07	SP80-3280	RB835486	3.73	6.67	2.05	700	Brazil						
Cultivar	F08	SP80-3280	RB835486	5.11	3.32	2.93	700	Brazil						
Cultivar	F09	SP80-3280	RB835486	6.90	4.89	2.45	700	Brazil						
Cultivar	F10	SP80-3280	RB835486	7.37	5.70	2.08	700	Brazil						
Cultivar	F31-962	Co-281	CP27-108	5.11	7.08	6.43	700	Formosa (Taiwan)						
Cultivar	F36-819	F31-962	POJ2878	5.85	2.92	5.19	700	Formosa (Taiwan)						

Genus/ species	cultivar/ accession	Parent 1	Parent 2	Loci 11K15	Loci 15015	Loci 44D02	Real-Time PCR system used	Provenance	Sucrose content	Fibre content	Smut	Brown rust	Leaf scald	Sugarcane Mosaci Virus (SGMV)
Cultivar	H53-3989	H48-3717	?	2.89	6.30	4.40	700	Hawaii						
Cultivar	H59-1966	H50-676	H49-3646	5.28	3.84	4.26	700	Hawaii						
Cultivar	IAC50-134	Co419	Co285	2.93	2.42	7.03	700	Brazil	2					
Cultivar	IAC51-205	POJ2878	?	4.53	0.82	6.05	700	Brazil			1			
Cultivar	IAC52-150	Co419	Co285	5.68	5.37	6.02	700	Brazil			4			
Cultivar	IAC64-257	Co419	IAC49-131	4.30	6.87	4.79	700	Brazil	2		1			
Cultivar	IAC68-12	Co419	IAC52-179	6.34	7.13	4.93	700	Brazil			4			
Cultivar	IAC82-3092	CB41-76	IAC68-12	6.35	4.83	6.36	700	Brazil			1	1		
Cultivar	IAC83-4157	IAC68-12	SP70-1143	5.52	4.64	5.71	700	Brazil						
Cultivar	IAC86-2210	CP52-48	Co798	4.41	4.58	0.02	700	Brazil			5	1		
Cultivar	IAC91-1099	RB785148	?	4.88	4.28	3.30	700	Brazil	2					
Cultivar	MIDA			5.48	4.83	3.93	ViiA							
Cultivar	NA56-79	Co419	Co419	3.35	2.50	5.19	700	Argentina			intermediaria			
Cultivar	Nco310	Co421	Co312	5.78	5.56	3.52	700	India						
Cultivar	POJ2878	POJ2364	EK28	4.40	3.84	7.01	700	Java						
Cultivar	Q124	Nco310	QN54-7096	4.04	3.85	4.94	ViiA	Queensland, Australia			6	1	1	7
Cultivar	Q142			4.57	2.17	3.66	ViiA	Queensland, Australia			4		2	1
Cultivar	Q165			5.60	4.59	3.53	ViiA	Queensland, Australia			8		3	1
Cultivar	Q172	Q99	H49-3666	4.00	5.16	5.79	ViiA	Queensland, Australia			4	unknown	1	
Cultivar	Q173			4.10	5.74	5.16	ViiA	Queensland, Australia			4		2	5
Cultivar	Q186			6.60	3.36	5.77	ViiA	Queensland, Australia						
Cultivar	Q193	CP51-21	Q121	1.86	5.76	5.76	ViiA	Queensland, Australia			9	unknown	1	4
Cultivar	Q198			2.65	5.16	3.57	ViiA	Queensland, Australia						
Cultivar	Q200	QN23-1700	QN66-2008	5.22	2.23	2.24	ViiA	Queensland, Australia			1	1	1	1
Cultivar	Q201			2.54	1.77	4.76	ViiA	Queensland, Australia			7		2	8
Cultivar	Q208	Q135	QN61-1232	5.57	6.94	1.95	ViiA	Queensland, Australia			4	1	1	1
Cultivar	Q212	Q138	H56-752	7.63	8.10	1.70	ViiA	Queensland, Australia			1	6	1	
Cultivar	Q217			4.05	4.51	4.04	ViiA	Queensland, Australia			8		1	1

Genus/ species	cultivar/ accession	Parent 1	Parent 2	Loci 11K15	Loci 15015	Loci 44D02	Real-Time PCR system used	Provenance	Sucrose content	Fibre content	Smut	Brown rust	Leaf scald	Sugarcane Mosaci Virus (SGMV)
Cultivar	Q221			5.54	5.15	6.10	ViiA	Queensland, Australia			7		5	1
Cultivar	Q99			5.41	5.03	5.90	ViiA	Queensland, Australia						
Cultivar	R570	H32-8560	R445	1.70	2.03	3.59	700	Reunion						
Cultivar	RB721012	Co331	?	4.44	1.54	4.69	700	RIDESA, Brazil	2	3	1	6	1	1
Cultivar	RB72199	NCo334	?	2.65	1.92	7.18	700	RIDESA, Brazil						
Cultivar	RB72454	CP53-76	?	3.59	2.97	6.13	700	RIDESA, Brazil	2	3	1	1	2	1
Cultivar	RB725053	Co775	?	5.06	2.73	5.66	700	RIDESA, Brazil						
Cultivar	RB725828	NA56-79	?	2.61	1.52	4.95	700	RIDESA, Brazil	2	3	5	7	2	6
Cultivar	RB732577	NCo376	?	4.06	3.63	3.85	700	RIDESA, Brazil	2	3	?	?	1	?
Cultivar	RB735200	Co331	?	4.01	1.54	5.92	700	RIDESA, Brazil						
Cultivar	RB735220	CB61-99	?	5.07	5.05	5.52	700	RIDESA, Brazil	2	3	1	1	1	1
Cultivar	RB735275	IAC49-131	?	1.35	8.05	5.38	700	RIDESA, Brazil	4	3	2	1	2	2
Cultivar	RB739359	IANE55-34	?	7.54	1.45	5.44	700	RIDESA, Brazil	2					
Cultivar	RB739735	CB52-179	?	3.91	3.24	3.60	700	RIDESA, Brazil	2	3	5	3	2	
Cultivar	RB75126	C278	?	3.12	1.88	3.61	700	RIDESA, Brazil	4	3	1	1	1	?
Cultivar	RB765418	M253/48	?	4.25	4.30	3.15	700	RIDESA, Brazil	2	3	1	1	1	3
Cultivar	RB785148	IAC47-31	?	4.81	3.60	4.44	700	RIDESA, Brazil	4	3	5	1	2	3
Cultivar	RB825317	L60-14	CB47-355	2.14	5.22	4.69	700	RIDESA, Brazil						
cultivar	RB825336	H53-3989	?	2.31	6.49	2.38	700	RIDESA, Brazil	4	1	1	1	1	1
Cultivar	RB835019	RB72454	NA56-79	2.35	2.56	4.22	700	RIDESA, Brazil	2	1	5	1	1	1
cultivar	RB835089	RB72454	NA56-79	3.81	1.40	7.44	700	RIDESA, Brazil	4	3	1	1	3	1
Cultivar	RB835205	Co740	?	5.27	2.80	1.98	700	RIDESA, Brazil						
Cultivar	RB845197	RB72454	SP70-1143	2.06	3.62	5.13	700	RIDESA, Brazil	4	3	5	3	2	3
cultivar	RB845210	RB72454	SP70-1143	3.87	2.89	6.16	700	RIDESA, Brazil	2	3	1	1	1	1
cultivar	RB845257	RB72454	SP70-1143	2.72	3.61	5.15	700	RIDESA, Brazil	2	3	1	1	1	1
Cultivar	RB855002	SP70-1143	RB72454	2.50	4.25	5.69	700	RIDESA, Brazil						
cultivar	RB855035	L60-14	SP70-1284	5.18	2.61	3.81	700	RIDESA, Brazil	2	3	1	5	1	1
cultivar	RB855036	RB72454	SP70-1143	3.65	5.58	5.23	700	RIDESA, Brazil	4	3	1	1	1	1

Genus/ species	cultivar/ accession	Parent 1	Parent 2	Loci 11K15	Loci 15015	Loci 44D02	Real-Time PCR system used	Provenance	Sucrose content	Fibre content	Smut	Brown rust	Leaf scald	Sugarcane Mosaci Virus (SGMV)
Cultivar	RB855077	SP70-1143	TUC71-7	1.35	6.04	3.55	700	RIDESA, Brazil						
Cultivar	RB855113	SP70-1143	RB72454	4.13	4.19	4.69	700	RIDESA, Brazil	2	5	1	1	1	1
cultivar	RB855156	RB72454	TUC71-7	1.15	3.00	3.86	700	RIDESA, Brazil	2	5	1	1	1	1
Cultivar	RB855350	RB72454	?	4.62	3.04	3.48	700	RIDESA, Brazil						
cultivar	RB855453	TUC71-7	?	1.60	3.29	4.15	700	RIDESA, Brazil	1	3	1	1	1	1
Cultivar	RB855463	RB72454	?	3.93	3.77	5.64	700	RIDESA, Brazil	2	3	1	1	1	1
Cultivar	RB855465	RB72454	?	1.25	3.31	2.20	700	RIDESA, Brazil						
Cultivar	RB855511	SP71-1406	?	4.78	2.07	5.16	700	RIDESA, Brazil	4	3	1	2	1	3
Cultivar	RB855563	TUC71-7	SP70-1143	3.35	3.33	2.88	700	RIDESA, Brazil	2	3	1	3	1	1
Cultivar	RB855595	SP70-1143	TUC71-7	4.36	4.64	2.31	700	RIDESA, Brazil						
cultivar	RB925211	RB855206	?	3.50	2.18	4.20	700	RIDESA, Brazil	4	2	1	1	1	1
cultivar	RB925268	RB855511	?	6.18	4.08	5.47	700	RIDESA, Brazil	4	3	1	1	1	5
Cultivar	RB92579	RB75126	RB72199	5.93	1.76	5.93	700	RIDESA, Brazil	2	3	?	3	1	?
cultivar	RB935744	RB835089	RB765418	6.91	3.62	5.62	700	RIDESA, Brazil	4	3	1	1	1	5
cultivar	RB965902	RB855536	RB855453	5.64	0.60	5.70	700	RIDESA, Brazil	2	3	1	1	1	1
cultivar	RB965917	RB855453	RB855536	2.61	4.31	3.48	700	RIDESA, Brazil	2	3	1	1	1	1
Cultivar	RB966928	RB855156	RB815690	4.83	2.48	5.22	700	RIDESA, Brazil	4	3	5	3	2	3
Cultivar	SP70-1078	IAC48-65	?	5.00	2.38	2.09	700	CTC, Brazil			1			
Cultivar	SP70-1143	IAC48-65	?	3.48	7.71	6.02	700	CTC, Brazil			1	1		
Cultivar	SP70-1284	CB41-76	?	4.64	5.43	3.29	700	CTC, Brazil			1			
Cultivar	SP70-1423	CB41-76	?	4.20	5.17	3.36	700	CTC, Brazil						
Cultivar	SP70-3370	CP53-17	?	4.99	3.51	4.22	700	CTC, Brazil			1			
Cultivar	SP71-6163	NA56-79	?	6.01	6.59	5.05	700	CTC, Brazil						
Cultivar	SP71-6949	NA56-79	?	5.04	4.10	4.57	700	CTC, Brazil						
Cultivar	SP79-1011	NA56-79	Co775	4.99	3.31	2.24	700	CTC, Brazil	2	3	3	1		
Cultivar	SP79-2233	H56-2954	?	4.55	4.02	2.55	700	CTC, Brazil	2					
Cultivar	SP79-2312	SP71-6106	?	5.44	6.33	2.47	700	CTC, Brazil						
Cultivar	SP79-2313	SP71-6106	?	4.71	6.45	2.52	700	CTC, Brazil						

Genus/ species	cultivar/ accession	Parent 1	Parent 2	Loci 11K15	Loci 15015	Loci 44D02	Real-Time PCR system used	Provenance	Sucrose content	Fibre content	Smut	Brown rust	Leaf scald	Sugarcane Mosaci Virus (SGMV)
Cultivar	SP79-6134	H63-4644	?	5.85	7.65	0.00	700	CTC, Brazil						
Cultivar	SP79-6192	SP70-3518	?	3.85	5.36	0.00	700	CTC, Brazil	2					
Cultivar	SP80-1520	H48-3166	SP71-1088	3.85	5.67	5.03	700	CTC, Brazil						
Cultivar	SP80-1816	SP71-1088	H57-5028	6.40	3.18	4.37	700	CTC, Brazil	2	1	5	3	2	
Cultivar	SP80-1836	SP71-1088	H57-5028	7.59	5.30	5.22	700	CTC, Brazil						
Cultivar	SP80-1842	SP71-1088	H57-5028	6.19	2.81	5.79	700	CTC, Brazil	2		5			
Cultivar	SP80-3280	SP71-1088	H57-5028	5.13	3.92	4.43	700	CTC, Brazil	2	1	1	1	2	1
Cultivar	SP81-3250	CP70-1547	SP71-1279	5.77	3.77	6.71	700	CTC, Brazil	2	1	8	1	4	
Cultivar	SP83-5073	SP71-1406	SP71-1088	4.37	3.09	6.37	700	CTC, Brazil	2	1	4	4	1	1
Cultivar	SP89-1115	CP73-1547	?	4.28	5.82	0.00	700	CTC, Brazil	2	5	1	1	1	1
Cultivar	SP91-1049	SP80-3328	SP81-3250	5.09	7.28	4.81	700	CTC, Brazil	2	3	7	1	4	1
Cultivar	Tellus			3.86	5.28	2.33	ViiA							
Cultivar	Triton			4.75	2.31	5.67	ViiA		4					
Cultivar	Trojan	Co270	S. officinarum	5.08	3.15	5.93	ViiA							
Cultivar	TUC71-7	CP52-68	CP62-258	1.22	2.70	2.36	700	Argentina						
Erianthus	Hainan 92-108			0.00	0.00	0.00	ViiA	China						
Erianthus	Hainan 92-79			0.00	0.00	0.00	ViiA	China						
Erianthus	Hainan 92-80			0.00	0.00	0.00	ViiA	China						
Erianthus	Sichuan 92-19			0.00	0.00	0.00	ViiA	China						
Erianthus	Yu 83-198			0.00	0.00	0.00	ViiA	China						
Miscanthus	Barola			0.00	0.00	0.00	ViiA	Indonesia						
Miscanthus	Kamanofi			0.00	0.00	0.00	ViiA	Indonesia						
Miscanthus	Komperi			0.00	0.00	0.00	ViiA	Indonesia						
Miscanthus	Ofare			0.00	0.00	0.00	ViiA	Indonesia						
Miscanthus	Teka			0.00	0.00	0.00	ViiA	Indonesia						
S. barberi	Chunnee			0.43	1.44	0.00	700							
S. edule	NG57-164			0.00	3.56	0.00	ViiA	New Guinea						
S. edule	NH70-0339			0.00	2.33	0.00	ViiA	New Hebrides						

Genus/ species	cultivar/ accession	Parent 1	Parent 2	Loci 11K15	Loci 15015	Loci 44D02	Real-Time PCR system used	Provenance	Sucrose content	Fibre content	Smut	Brown rust	Leaf scald	Sugarcane Mosaci Virus (SGMV)
S. edule	SE15			0.00	8.55	0.00	ViiA							
S. officinarium	Badilla			6.33	6.28	5.01	700	New Guinea						
S. officinarium	Black Tanna			2.64	6.92	4.90	ViiA							
S. officinarium	Creoula			4.22	3.21	3.03	700							
S. officinarium	G German			3.57	4.69	4.44	700							
S. officinarium	IEIE			1.91	2.01	2.31	ViiA	Hawaii						
S. officinarium	IJ76-514			2.66	7.33	4.68	ViiA	Irian Jaya						
S. officinarium	IK76-35			3.45	3.15	2.70	ViiA	Kalimantan						
S. officinarium	IM76-237			2.12	2.59	3.04	ViiA	Maluku, Indonesia						
S. officinarium	Korpi			4.21	3.89	3.48	ViiA	New Guinea						
S. officinarium	NG21-3			7.82	6.38	2.65	ViiA	New Guinea						
S. officinarium	NG21-5			3.99	1.27	5.94	ViiA	New Guinea						
S. robustum	IJ76-416			0.00	1.68	0.00	ViiA	Irian Jaya						
S. robustum	IJ76-422			0.00	4.25	0.00	ViiA	Irian Jaya						
S. robustum	IJ76-507			0.00	0.00	0.00	ViiA	Irian Jaya						
S. robustum	IJ76-534			0.00	2.80	0.00	ViiA	Irian Jaya						
S. robustum	IM76-229			0.00	0.00	0.00	ViiA	Maluku, Indonesia						
S. robustum	IS76-184			0.00	7.15	0.00	ViiA	Sulawesi, Indonesia						
S. robustum	MOL-4200			0.00	6.56	0.00	ViiA							
S. robustum	MOL-4943			0.00	7.47	0.00	ViiA							
S. robustum	NG47-208			0.00	2.84	0.00	ViiA	New Guinea						
S. robustum	T.T. Tengarron			0.00	7.22	0.00	ViiA							
S. sinense	Maneira			2.96	7.90	4.40	700							
S. spontaneum	Coimbatore			0.00	0.00	0.00	ViiA	Coimbatore, India						
S. spontaneum	IK76-49			0.00	0.00	0.00	ViiA	Kalimantan						
S. spontaneum	IS76-132			0.00	0.00	0.00	ViiA	Sulawesi, Indonesia						
S. spontaneum	Mandalay			0.00	0.00	0.00	ViiA							
S. spontaneum	Rellegadi			0.00	0.00	0.00	ViiA							

Genus/ species	cultivar/ accession	Parent 1	Parent 2	Loci 11K15	Loci 15015	Loci 44D02	Real-Time PCR system used	Provenance	Sucrose content	Fibre content	Smut	Brown rust	Leaf scald	Sugarcane Mosaci Virus (SGMV)
S. spontaneum	Saigon			0.00	0.00	0.00	ViiA							
S. spontaneum	SES231			0.00	0.00	0.00	ViiA							

Supplementary Table S1: Samples examined;species; cultivar/accession number; parentage; proportion of alleles at the three loci. BAC SCHRBa_011_K15 (scIvana 1.1), BAC SCHRBa_044_D02 (scIvana 1.2) and BAC SCHRBa_015_O15 (scIvana 1.4), with the scIvana element present; Real-Time system used; provenance; sucrose content, fibre content and susceptibility to smut, brown rust, leaf scald and sugarcane mosaic virus. Estimates of the ratio of alleles with the elements to alleles without the element were transformed into a total of 10. Only the value for the presence of the element is shown. Data taken mainly from (Cesnik and Miocque, 2004; Aitken *et al.* , 2006; *Variedades RB de cana-de-acúcar* , 2008, *Catálogo nacional de variedades "RB" de cana-de-acúca* , 2010; Landell and Bressiani, 2008; Santos, 2008; Cheavegatti-Gianotto *et al.* , 2011). Traits are shown as a scale: Sucrose content: 1, very high; 2, high; 3, good; 4, average; Fibre content: 1, high; 2, average-high; 3, average; 4, good; 5, low; Smut: 1, resistant; 2, highly tolerant; 3, moderately tolerant; 4, intermediate-resistant; 5, tolerant; 6, intermediate-susceptible; 7, moderately-susceptible; 8, susceptible; 9, highly-susceptible; Brown rust: 1, resistant; 2, highly-tolerant; 3, tolerant; 4, intermediate; 5, intermediate-average; Leaf-scald; 1, resistant; 2, tolerant; 3, intermediate-average; 4, average; Sugarcane mosaic virus: 1, resistant; 2, highly tolerant; 3, tolerant; 4, intermediate-resistant; 5, intermediate-average; 6, moderately-susceptible; 7, susceptible.

loci	transposable element	sequence	final concentration (nM)
SHCRBa_44D02 - scIvana present	scIvana1.2		
forward primer		ATATGAAAGAGCAGTCGCCTGGGT	300
reverse primer		AGTCTCATGGCCTCTGCAAACTGT	300
probe		FAM-TCCCTGGCTAGGAAGAAACACTCTGT-BKFQ	250
SHCRBa_44D02 - scIvana absent	scIvana1.2		
forward primer		AACGAGTTGCCGTAGACGGATGTA	300
reverse primer		TCTTCAATTCTGCCCTGTTCGGCT	50
probe		FAM-CCCAAGGATAATAAATGGCTGGGCCT-BkFQ	250
SHCRBa_15015 -scIvana present	scIvana1.4		
forward primer		ATATGAAAGAGCAGTCGCCTGGGT	900
reverse primer		ACACATTATGACTCCTGCCTGCGA	900
probe		FAM-AGCTGGAGCTGCATCTAGAACCTAAA-BKFQ	250
SHCRBa_15015 -scIvana absent	scIvana1.4		
forward primer		AACAGGACTGTGGAGCAGGTAGAT	900
reverse primer		ACACATTATGACTCCTGCCTGCGA	900
probe		FAM-AGCTGGAGCTGCATCTAGAACCTAAA-BKFQ	250
SHCRBa_11K15 - scIvana present	scIvana1.1		
forward primer		ATATGAAAGAGCAGTCGCCTGGGT	500
reverse primer		AGCGTTTAGTGGAAAGTGTTGCCC	500
probe		FAM-CTGTACCTCCAACACTATCGAGTCGACT-BKFQ	250
SHCRBa_11K15 - scIvana absent	scIvana1.1		
forward primer		AACAGAACACTGGTAGCAAGA	500
reverse primer		TAGTGGAAAGTGTTGCCCTAAA	500
probe		FAM-GAGAAATGCTGATTCTTATCCGCTGCTCT-BKFQ	250

Supplementary Table S2. Sequences and concentrations of primers and probes used. The primer concentrations are those used in Brazil on the Applied Biosystems 7300 Real-Time PCR system. In Australia, all primers were used at a final concentration of 500nm and reactions were run on an Applied Biosystems ViiA 7 Real-Time PCR system.

Trait	Group 1[b] scale[a]	%	Group 2 scale	%
Sucrose content	2	100%	1	3%
			2	63%
			4	34%
Fibre content	3	75%	1	18%
	5	25%	2	3%
			3	73%
			5	6%
Smut	1	58%	1	56%
	3	8%	2	2%
	4	8%	4	10%
	7	8%	5	20%
	8	17%	6	2%
			7	5%
			8	2%
			9	2%
Brown rust	1	75%	1	73%
	3	13%	2	3%
	6	13%	3	12%
			4	3%
			5	3%
			6	3%
			7	3%
Leaf scald	1	78%	1	63%
	3	11%	2	30%
	4	11%	3	3%
			4	3%
			5	3%
SGMV	1	88%	1	64%
	3	13%	2	3%
			3	12%
			4	3%
			5	9%
			6	3%
			7	3%
			8	3%

Supplementary Table S4. For each group[b] identified by phylogenetic analysis (Supplementary Figure S2), the percentage of cultivars from the Australian and Brazilian breeding programs (RB, SP, Q-canes and the F series) that fall into each trait scale. [a]scale: please see legend for Supplementary Table S1.

BAC			Grass Genomes											
number	location begin	location end	Genome	chrom	location begin	location end	locus	description	PANTHER	PFAM	PFAM	PFAM	KOG	KEGGORTH
**SHRBa_011_K15**														
5` to BAC														
			Sorghum bicolor	8	42417857	42418225	Sobic.008G105700							
BAC begin														
	15632	20539					SHRBa_011_K15.3	elongation factor Tu						
			Sorghum bicolor	8	42533513	42539374	Sobic.008G106000	Putative uncharacterized		PF00009	PF03144	PF11987	KOG1144	
			Oryza sativa	12	19199289	19205185	LOC_Os12g31880	translation initiation factor		PF00009	PF03144	PF11987		
	20770	23067					SHCRBa_011_K15.4	Ureide Permease						
			Sorghum bicolor	8	42539020	42541559	Sobic.008G106100	Putative uncharacterized		PF07168				
			Oryza sativa	12	19161734	19165432	LOC_Os12g31890	Ureide Permease		PF07168				
	97619	98821					SHCRBa_011_K15.13	pinoresinol_reductase_1						
			Sorghum bicolor	8	42768091	42771347	Sobic.008G106700	similar to isoflavone reductase	PTHR14194	PF05368				
			Zea mays	3	135111818	135133286	GRMZM2G326116	pinoresinol reductase	PTHR14194	PF05368				K00224
	101531	106814					scIvana1.1							
BAC end														
3` to BAC														
				8	42761066	42761821	Sobic.008G106600	hypothetical						
**SHCRBa_044_D02**														
5` to BAC			*Sorghum bicolor*	7	61280504	61283646	Sobic.007G192600	similar to FACT complex subunit SPT16	PTHR13980	PF00557	PF08512	PF08644	KOG1189	
			Oryza sativa	8	19314778	19318231	LOC_Os08g31240	FACT complex subunit SPT16 putative expressed	PTHR13980	PF00557		PF08644	KOG1189	
			Sorghum bicolor	7	61288124	61291009	Sobic.007G192700	similar to Os05g0373700	PTHR21713	PF00627	PF01849		KOG2239	K03626
			Zea mays	1	60165434	60167726	GRMZM2G000923	Nascent polypeptide-associated complex (NAC) alpha/source	PTHR21713		PF01849		KOG2239	K03626
			Zea mays				GRMZM5G877019	hypothetical						

BAC			Grass Genomes											
number	location		Genome	chrom	location		locus	description	PANTHER	PFAM	PFAM	PFAM	KOG	KEGGORTH
	begin	end			begin	end								
			Sorghum bicolor	7	61293371	61296157	Sobic.007G192800	similar to Nuclear transport factor 2	PTHR12612	PF02136			KOG2104	
			Oryza sativa	8	26535057	26537389	LOC_Os08g42000	nuclear transport factor putative expressed	PTHR12612	PF02136			KOG2104	
			Zea mays	1	194524205	194526962	GRMZM2G407249	hypothetical	PTHR12612	PF02136			KOG2104	
			Sorghum bicolor	7	61296187	61311504	Sobic.007G192900	hypothetical	PTHR11945	PF00319				
			Oryza sativa	8	26518976	26529016	LOC_Os08g41960	OsMADS37 MADS-box family gene with MIKC* type-box expressed	PTHR11945	PF00319				
							GRMZM2G443517	hypothetical						
			Sorghum bicolor	7	61312378	61316071	Sobic.007G193100	hypothetical						
			Sorghum bicolor	7	61340052	61345722	Sobic.007G193200	similar to Methylthioadenosine/S-adenosyl nucleosidase	PTHR21234	PF01048				K01244
			Zea mays	1	194243577	194252745	GRMZM2G171111	methylthioadenosine nucleosidase 1	PTHR21234	PF01048				K01244
			Sorghum bicolor	7	61349792	61357683	Sobic.007G193300	similar to putative MADS-domain transcription factor	PTHR11945	PF00319	PF01486		KOG0014	
			Oryza sativa	8	26507180	26512566	LOC_Os08g41950	OsMADS7 - MADS-box family gene with MIKCc type-box	PTHR11945	PF00319	PF01486		KOG0014	
			Zea mays	1	194046502	194054556	GRMZM2G159397	K-box region and MADS-box transcription factor	PTHR11945	PF00319	PF01486		KOG0014	
			Sorghum bicolor				Sobic.007G193400	hypothetical						
			Sorghum bicolor	7	61367404	61373899	Sobic.007G193500	similar to Teosinte glume architecture 1		PF03110				
			Oryza sativa	8	26501167	26506218	LOC_Os08g41940	OsSPL16 - SBP-box gene family member		PF03110				
BAC begin														
	4567	6679					SHCRBa_044_D02.1	glycosylphosphatidylinositol						
			Sorghum bicolor	7	61256609	61258839	Sobic.007G192200	similar to Lipid transfer protein-like		PF00234				
			Oryza sativa	8	26554156	26557080	LOC_Os08g42040	LTPL80 - Protease inhibitor/seed storage/LTP family expressed		PF00234				
			Zea mays	1	194690454	194692196	GRMZM2G083725	hypothetical		PF00234				
	9728	15016					scIvana1.2							
	15181	18458					SHCRBa_044_D02.4	emp24gp25Lp24_familyGOLD_family						
			Sorghum bicolor	7	61251598	61254964	Sobic.007G192100	similar to Coated vesicle membrane protein-like	PTHR22811	PF01105			K0G1692	

BAC			Grass Genomes											
number	location		Genome	chrom	location		locus	description	PANTHER	PFAM	PFAM	PFAM	KOG	KEGGORTH
	begin	end			begin	end								
			Oryza sativa	8	26558844	26562158	LOC_Os08g42050	emp24/gp25L/p24 family protein putative expressed	PTHR22811	PF01105			K0G1692	
			Zea mays	1	194693263	194696454	GRMZM2G083551	emp24/gp25L/p24 family/GOLD family	PTHR22811	PF01105			K0G1692	
	27327	33102					SHCRBa_044_D02.7	ACT_domain_repeat_4						
			Sorghum bicolor	7	61238225	61241528	Sobic.007G192000	similar to Os08g0533300	PTHR13734	PF01842				
			Oryza sativa	8	26572162	26574946	LOC_Os08g42080	ACR5 putative expressed	PTHR13734	PF01842				
			Zea mays	1	194707434	194710731	GRMZM2G083538	ACT domain/source	PTHR13734	PF01842				
							SHCRBa_044_D02.9	C-terminal region of a signal						
	63457	67106					SHCRBa_044_D02.11	ACT_domain_repeat_6						
			Sorghum bicolor	7	61219428	61222940	Sobic.007G191900	similar to putative uncharacterized	PTHR13734	PF01842				
			Oryza sativa	8	26601165	26605582	LOC_Os08g42100	ACT domain containing protein expressed	PTHR13734	PF01842				
			Zea mays	1	194761999	194765199	GRMZM2G135617	hypothetical	PTHR13734	PF01842				
	67250	74900					SHCRBa_044_D02.12	Ribosomal protein L7AeL30eS12eGadd45 family						
							Conserved but not predicted in Sorghum							
			Oryza sativa	8	14340459	14342940	LOC_Os08g23710	Ribosomal protein L7Ae putative expressed	PTHR23105	PF01248			KOG3166	KO2936
			Zea mays	1	194764577	194767711	GRMZM2G135654	Ribosomal protein L7Ae/L30e/S12e/Gadd45 family/source	PTHR23105	PF01248			KOG3166	KO2936
			Zea mays				GRMZM2G135733	hypothetical						
BAC end														
3` to BAC			*Sorghum bicolor*	7	61202234	61206647	Sobic.007G191700	similar to Cation exchanger-like		PF09753				
			Oryza sativa	8	26606691	26609538	LOC_Os08g42110	expressed protein	PTHR13050	PF09753				
			Zea mays	1	194767910	194771938	GRMZM2G436319	Membrane fusion protein Use1		PF09753				
			Sorghum bicolor	7	61194917	61201356	Sobic.007G191600	similar to Os11g0311300	PTHR11097	PF01138	PF03725		KOG1613	K12586

BAC			Grass Genomes											
number	location		Genome	chrom	location		locus	description	PANTHER	PFAM	PFAM	PFAM	KOG	KEGGORTH
	begin	end			begin	end								
			Zea mays	1	194789367	194798364	GRMZM5G886044	3'-5'-exoribonuuclease family	PTHR11097	PF01138	PF03725		KOG1613	K12586
			Sorghum bicolor				Sobic.007G191500	hypothetical						
			Zea mays				GRMZM2G075042	hypothetical						
			Sorghum bicolor	7	61189971	61191064	Sobic.007G191400	similar to Putative uncharacterized		PF02519				
			Sorghum bicolor	7	61181579	61189532	Sobic.007G191300	hypothetical	PTHR24420	PF00069			KOG1187	
							Truncated, conserved but not predicted in Maize							
			Sorghum bicolor	7	61176997	61180220	Sobic.007G191200	similar to Disease resistant allele XA13	PTHR10791	PF03083			KOG1623	
			Oryza sativa	8	26725898	26728795	LOC_Os08g42350	nodulin MtN3 family protein, putative expressed	PTHR10791	PF03083			KOG1623	
			Zea mays	1	194965437	194968345	GRMZM2G368827	senescence-associated gene 29	PTHR10791	PF03083			KOG1623	
			Zea mays	1	194965843	194968109	GRMZM5G875082	hypothetical						
			Sorghum bicolor	7	61152794	61156551	Sobic.007G191100	similar to Os09g0508300	PTHR22883	PF01529			KOG1311	
			Oryza sativa	8	26744794	26748485	LOC_Os08g42370	zinc finger DHHC domain-containing protein putative expressed	PTHR22883	PF01529			KOG1311	
			Zea mays	1	195026666	195030944	GRMZM2G068657	DHHC-type zinc finger family	PTHR22883	PF01529			KOG1311	
			Sorghum bicolor	7	61151778	61152515	Sobic.007G191000	hypothetical						
			Sorghum bicolor	7	61142929	61144881	Sobic.007G190900	hypothetical						
			Sorghum bicolor	7	61139146	61142697	Sobic.007G190800	similar to putative uncharacterized	PTHR21535	PF02953			KOG3489	
			Oryza sativa	8	26749936	26751013	LOC_Os08g42380	mitochondrial import inner membrane translocase expressed	PTHR21535	PF02953			KOG3489	
			Zea mays	1	195074393	195076645	GRMZM2G075003	translocase inner membrane subunit 8	PTHR21535	PF02953			KOG3489	
			Sorghum bicolor	7	61132957	61140064	Sobic.007G190700	similar to PHO85-like	PTHR22958	PF03009			KOG2421	
			Oryza sativa	8	26751370	26754564	LOC_Os08g42390	glycerophosphoryl diester phosphodiesterase family expressed	PTHR22958	PF03009			KOG2421	

BAC			Grass Genomes											
number	location		Genome	chrom	location		locus	description	PANTHER	PFAM	PFAM	PFAM	KOG	KEGGORTH
	begin	end			begin	end								
			Zea mays	1	195179488	195184434	GRMZM2G064962	PLC-like phosphodiesterase superfamily	PTHR22958	PF03009			KOG2421	
			Sorghum bicolor	7	61116847	61120167	Sobic.007G190500	hypothetical						
			Oryza sativa	8	26766816	26770345	LOC_Os08g42400	no apical meristem protein putative expressed		PF02365				
			Zea mays	1	195218120	195221530	GRMZM2G054252	BAC domain/source		PF02365				
			Zea mays	1	195217815	195219618	GRMZM2G054383	hypothetical						
			Sorghum bicolor	7	61091240	61096923	Sobic.007G190400	hypothetical		PF10536				
			Sorghum bicolor	7	61084921	61089051	Sobic.007G190300	hypothetical						

Supplementary Table S3. Genomic neighbourhood of scIvana elements. The three BACs with the scIvana elements, SCHRBa_044_D02 (scIvana1.2), SCHRBa_011_K15 (scIvana1.1) SCHRBa_015_O15 (scIvana1.4) were masked and then screened against three grass genomes *S. bicolor* (v2.1), *Z. mays* (v6a) and *O. sativa* (v7.0). Genes were identified in the BACs and the three grass genomes. To identify putative genes in the sugarcane genome close to the BAC, genes in in *S. bicolor* , *Z. mays* and *O. sativa* were identified in the regions 100kb 5' and 3' to the BAC. Supplementary Table S3 shows the locus, description, chromosome, location of each gene and the functional annotation from the Panther, PFAM and KEGG databases (Kanehisa and Goto, 2000; Punta *et al.* , 2012; Mi *et al.* , 2013).

References

Aitken KS, Li J-C, Jackson P, Piperidis G, McIntyre CL. 2006. AFLP analysis of genetic diversity within *Saccharum officinarum* and comparison with sugarcane cultivars. *Australian Journal of Agricultural Research* **57**, 1167–1184.

Catálogo nacional de variedades "RB" de cana-de-acúca. 2010. Curitiba, Brazil: Rede Interuniversitária para o Desenvolvimento do Setor Sucroalcooleiro.

Cesnik R, Miocque J. 2004. Melhoramento da cana-de-açúcar. Brasília: Embrapa Informações Tecnológicas.

Cheavegatti-Gianotto A, de Abreu HMC, Arruda P, *et al.* 2011. Sugarcane (Saccharum X officinarum): A Reference Study for the Regulation of Genetically Modified Cultivars in Brazil. *Tropical plant Biology* **4**, 62–89.

Kanehisa M, Goto S. 2000. KEGG: kyoto encyclopedia of genes and genomes. *Nucleic acids research* **28**, 27–30.

Landell M, Bressiani J. 2008. Melhoramento genético, caracterização e manejo varietal. In: Dinardo-Miranda L, Vasconcellos A, Landelli M, eds. Cana-de-açúcar. Campinas, Sao Paulo, Brazil: Instituto Agronomico, 101–155.

Mi H, Muruganujan A, Thomas PD. 2013. PANTHER in 2013: modeling the evolution of gene function, and other gene attributes, in the context of phylogenetic trees. *Nucleic acids research* **41**, D377–D386.

Punta M, Coggill PC, Eberhardt RY, *et al.* 2012. The Pfam protein families database. *Nucleic acids research* **40**, D290–301.

Santos A. 2008. Doenças causadas por fungos. In: Dinardo-Miranda L, Vasconcellos A, Landelli M, eds. Cana-de-açúcar. Campinas, Sao Paulo, Brazil: Instituto Agronomico, 423–435.

Variedades RB de cana-de-acúcar. 2008. São Carlos, Brazil: Centro de Ciências Agrárias Universidade Federal de São Carlos (CCA-UFSCAR).
